# Quantifying global redundant fisheries trade to streamline seafood supply chains

**DOI:** 10.1371/journal.pone.0305779

**Published:** 2024-07-10

**Authors:** Caitlin D. Kuempel, Emma Arnett, Carissa J. Klein

**Affiliations:** 1 Coastal and Marine Research Centre, Australian Rivers Institute, School of Environment and Science, Griffith University, Nathan, Australia; 2 School of Environment, Centre for Biodiversity and Conservation Science, University of Queensland, Queensland, Australia; Stockholm University, SWEDEN

## Abstract

Seafood plays an important role in sustainably feeding the world and is one of the most traded food products globally. However sustainability improvements are often focused on its production (e.g., aquaculture, fishing) rather than trade. Here, we quantify the magnitude and extent of global ‘redundant two-way’ seafood trade–the exchange of the same quantity of the same taxonomic species between two countries–to examine its prevalence and potential implications across the seafood supply chain. We focused on wild-caught seafood trade and found that redundant two-way trade has increased by 43%, between 2000 and 2015, making up 3.2% (7.7 Mt) of global seafood trade during that period. Although most countries were involved in redundant two-way seafood trade (111 of 212 analyzed), the majority occurred between five trade partners: Canada and the United States (15%), Germany and the Netherlands (11.8%); Denmark and Sweden (10.6%); Germany and Denmark (7.1%); and France and Norway (7%). Nearly 50% of redundant trade is made up of just four species including Atlantic herring, Atlantic cod, Skipjack tuna and Atlantic mackerel. While deficiencies in global seafood trade data mask seasonal and product heterogeneity, redundant trade could have implications for meeting conservation and sustainable development goals. Future research should build upon these findings to explore specific environmental, economic, and social implications associated with redundant two-way trade to benefit producers and consumers within the seafood supply chain.

## Introduction

International trade has dramatically transformed the global economy over the past two centuries. Trade is an essential component of meeting many United Nations Sustainable Development Goals including ending hunger and achieving food security (SDG 2), however it can also decrease resilience of countries to supply shocks and result in substantial impacts on socioeconomic and environmental sustainability due to increased resource demand and decoupling of producers and consumers [1, 2]. Improving the sustainability of trade is of paramount importance for assessing progress towards and achieving sustainable development goals at national and international scales.

Sustainable trade creates economic value, reduces poverty and inequality, and preserves and reuses environmental resources [3]. Numerous environmental organisations have been advocating for more sustainable trade and the United Nations has developed several agendas to address environmental and socio-economic problems from trade (e.g., Conference on Trade and Development, Environment Programme, and Committee for Sustainable Development) [4]. The primary mechanisms to achieve sustainable trade are through the inclusion of environmental and social safeguards in international trade agreements (e.g., World Trade Organization and environmental provisions in preferential trade agreements), incorporation of social and environmental costs directly into trading operations, and removing trade barriers for environmental goods and services [5].

Seafood plays an important role in sustainably feeding the world [6, 7] and is one of the most highly traded foods [8]. Even though seafood is one of the most sustainable sources of animal protein [9], the production of seafood is associated with significant negative environmental and social impacts [10, 11]. As such, improving seafood sustainability has focused largely on production (e.g., aquaculture, fishing). Less research, however, has focused on improving the sustainability of seafood trade, a critical part of the supply chain. In some cases, seafood trade is likely to have negative environmental and social impacts, ranging from excessive greenhouse gas emissions and spread of invasive species associated with transportation [12] to supporting modern slavery [10]. Further, seafood trade allows for the displacement of environmental impacts far from the place of consumption [13] and makes environmental and social impacts more difficult to trace and manage [14].

**Fig 1:**
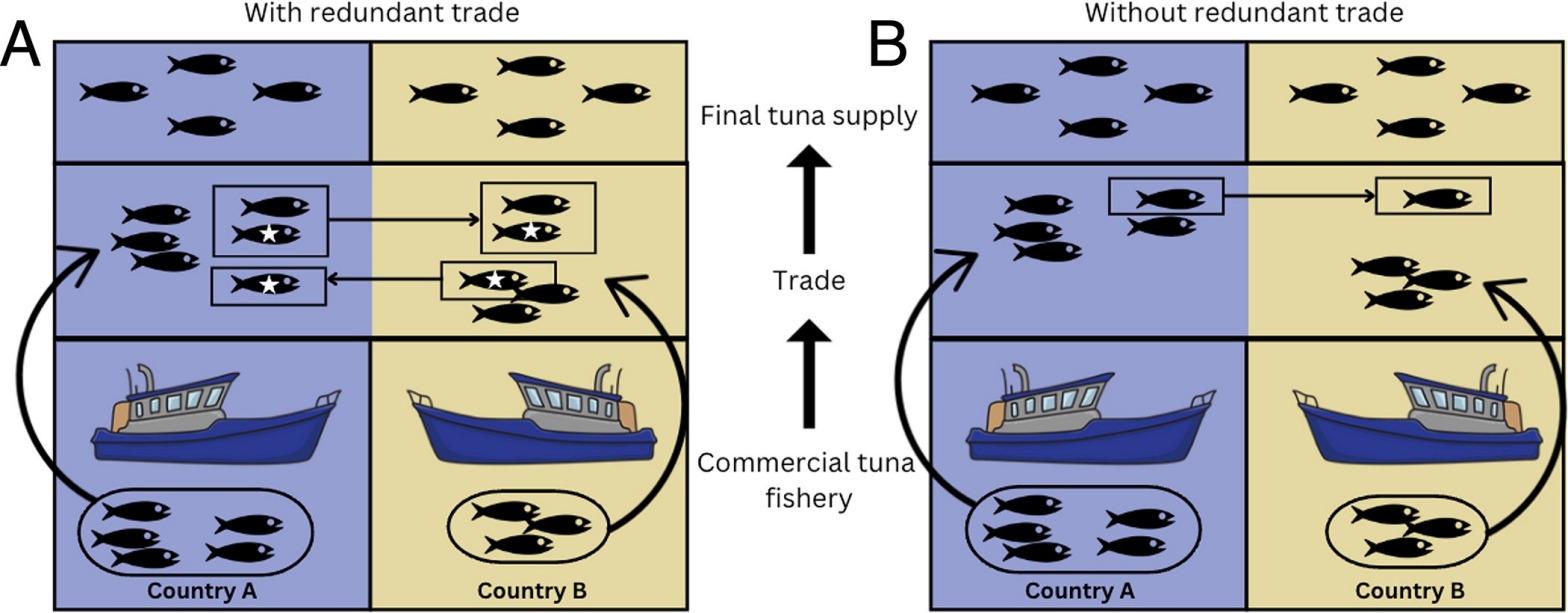
Hypothetical schematic explaining redundant two-way trade of wild-caught tuna between two countries. (A) Each country catches tuna, some of which is consumed within the country and some of which is traded (‘Trade’). If redundant two-way trade did not occur (B), both countries could still consume the same amount of tuna (‘Final tuna supply’). Fish indicated by white star icons in (A) represent redundant two-way trade. Vector images were sourced from pixabay.com from images produced before 2019, which fall under a creative commons zero license.

One way to streamline seafood trade is to eliminate exchanging the same quantity and type (e.g., species and/or product) of seafood between two countries (hereafter “redundant two-way trade”) [15, 16] (Fig 1). Although there are numerous socioeconomic and ecological factors that may explain redundant trade (e.g., seasonality, management restrictions, financial incentives), the reduction of redundant two-way trade could have social and environmental benefits. While two-way trade (i.e., intra-industry trade) of the same or similar goods in an industry is well documented (e.g., automobiles, agriculture) [17], and can be motivated by economic efficiencies, less is known about trade in identical commodities (in our case products of the same taxonomic species), particularly in the seafood sector. Here, we analyze global seafood trade data (excluding re-exports) to calculate the amount of redundant two-way trade between countries and the taxonomic species traded. We focus on wild-caught seafood products between 2000 and 2015. Data deficiencies in global seafood trade data currently prohibit further exploration beyond the annual and taxonomic species level (i.e., compared to seasonal and product differentiation), which introduces uncertainties in our results. This study determines the volume, extent, trade partners, and taxonomic species involved in redundant trade practices given best available data, which is the first step in understanding what, if any, sustainability gains could be made through the reduction of redundant trade.

## Methods

We used a global seafood trade database that estimates the annual volume of seafood imported by each country and its origin (i.e., exporting country) [18]. We chose to use this dataset because it identifies if the export product was produced through aquaculture or wild capture fisheries and categorizes if each record was a re-export (i.e., exported more than once). It also has the longest time series of trade data currently available. We excluded aquaculture records to reduce uncertainty around the comparability of species/products (i.e., is an aquaculture product equivalent to a wild-caught product?), and we excluded re-exported trade by only considering the initial exporting country and final importing country. This step eliminates double counting of trade and reduces consideration of processed goods that may occur due to re-exportation. We also excluded data that was not identifiable to the species level (e.g., ‘miscellaneous marine fishes’ or ‘elasmobranchii’, S1 and S2 Tables), resulting in data that described the trade of 272 species by 207 countries between 2000 and 2015. While this seafood trade dataset is the best currently available for our purposes, we recognize that the data is aggregated across time (year), location (country), and product form (taxonomic species), which masks heterogeneity and increases uncertainty in redundant trade estimates. Additionally, much of the data was not disaggregated to the species level and had to be excluded, which may underestimate our results.

Next, we quantified the level of trade of the same taxonomic species between two countries (hereafter “trade partners”), each year. We documented the identity, number, and volume (tonnes) of species involved in redundant two-way trade. Redundant trade (R_ij_) between country *i* and country *j* (*i* ≠ *j*) was calculated as:

$$R_{ij}=min(E_{ijs},E_{jis})\times 2$$ (1)

Where E_ijs_ is the amount (tonnes) of species, *s*, exported by country *i* to country *j* and E_jis_ is the amount (tonnes) of the same species, *s*, exported by country *j* to country *i*. For example, if country *i* exports 10 tonnes of species *s* to country *j*, and country *j* exports 5 tonnes of species *s* to country *i*, five tonnes of export could be avoided for both country *i* and country *j*, equating to 10 tonnes of redundant two-way trade in total (Fig 1).

## Results

**Fig 2:**
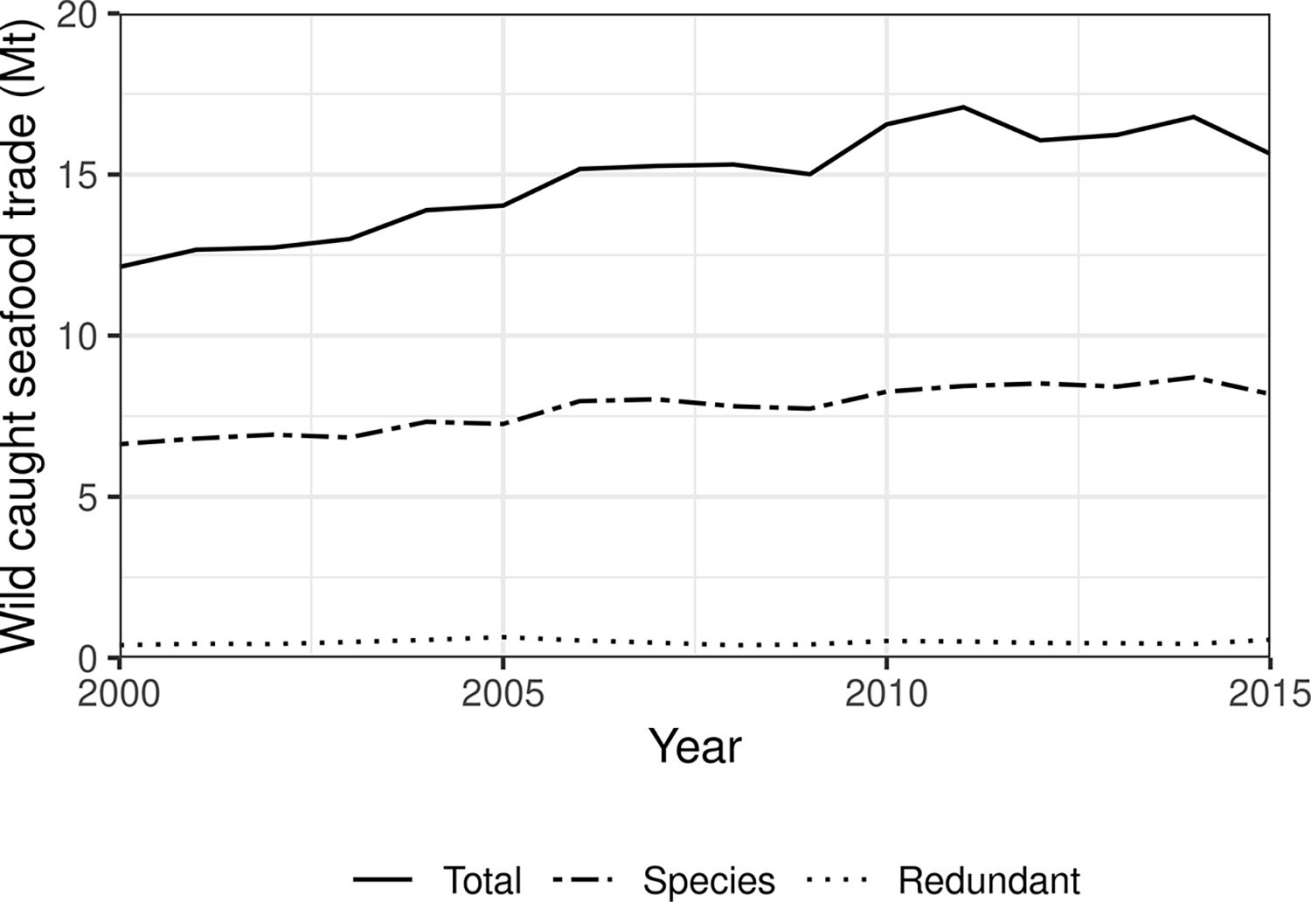
Wild-caught fisheries trade volume through time. Annual volume of wild-caught seafood traded globally in total (‘Total’), trade identified to species level (’Species’), and redundant trade (‘Redundant’) (2000–2015). Only traded seafood that could be identified to species level was used in our analysis of redundant trade.

Our data indicates 12.1–17.1 million tonnes (Mt) of wild-caught seafood were traded annually between 2000 and 2015 (Fig 2). Of this, 45.3–50.6% (6.6–8.7 Mt) was not identified to taxonomic species level and was omitted from redundant trade calculations (Fig 2 and S1 and S2 Tables), likely leading to lower estimates of redundant trade. We found that, depending on the year, 392–642 thousand tonnes (2.6–4.6%) was redundant two-way trade (Fig 2 and S3 Table), averaging 481.2 thousand tonnes per year. While values varied over time, redundant trade increased by 43.4% (~170 thousand tonnes) between 2000 and 2015, equating to 7.7 Mt of the 237.6 Mt of total global trade (3.2%).

**Fig 3:**
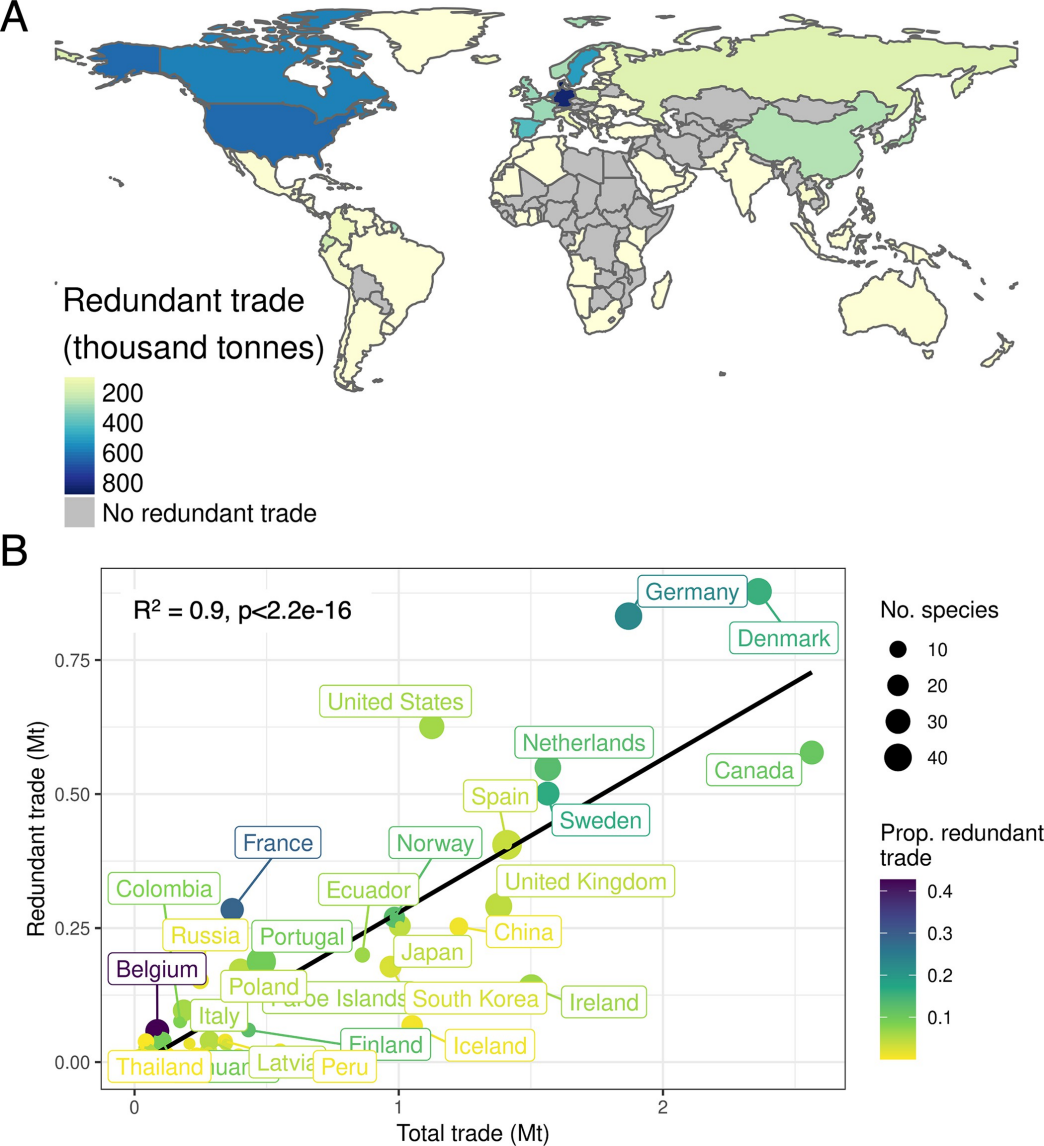
Country-level results of redundant and total trade. Volume of redundant trade (A) and the relationship between total trade and redundant trade (B) of wild-caught seafood exported from each country in total between 2000–2015. Colors in (B) represent the proportion of total trade that is redundant in each country and point size represents the number of species that were redundantly traded. Only countries in the top quartile of redundant trade are labelled. Map created in R using rworldmaps package (https://cran.r-project.org/web/packages/rworldmap/index.html) and Natural Earth data (http://www.naturalearthdata.com/).

Of the 212 countries that traded wild-caught seafood between 2000–2015, 111 countries (52%) participated in redundant trade (Fig 3A and S4 Table). The top 10 countries that contributed to redundant trade (by tonnes) were Denmark, Germany, the United States, Canada, the Netherlands, Sweden, Spain, the United Kingdom, France, and Norway, collectively accounting for 68% (5.2 Mt) of redundant trade across the entire period (Fig 3A). There were 10 countries where more than 10% of their total trade from 2000–2015 was classified as redundant (Fig 3B), including Belgium (43%; 57.9 thousand tonnes), France (29%; 284.4), Germany (23%; 831.9), Sweden (16%; 501.0), Denmark (16%; 877.9), Netherlands (13%; 549.3), Norway (13%; 270.2), Finland (12%; 59.9), Slovenia (12%; 0.56), and Canada (11%; 577.5 t). We found that the total amount of seafood exported from each country and amount classified as redundant trade were significantly positively correlated (R^2^ = 0.9 p < 2.2e-16; Fig 3B). Notably, the proportion of trade identified to species level (S5 Table) and amount of redundant trade was also positively correlated (R^2^ = 0.31 p = 0.0008).

**Fig 4:**
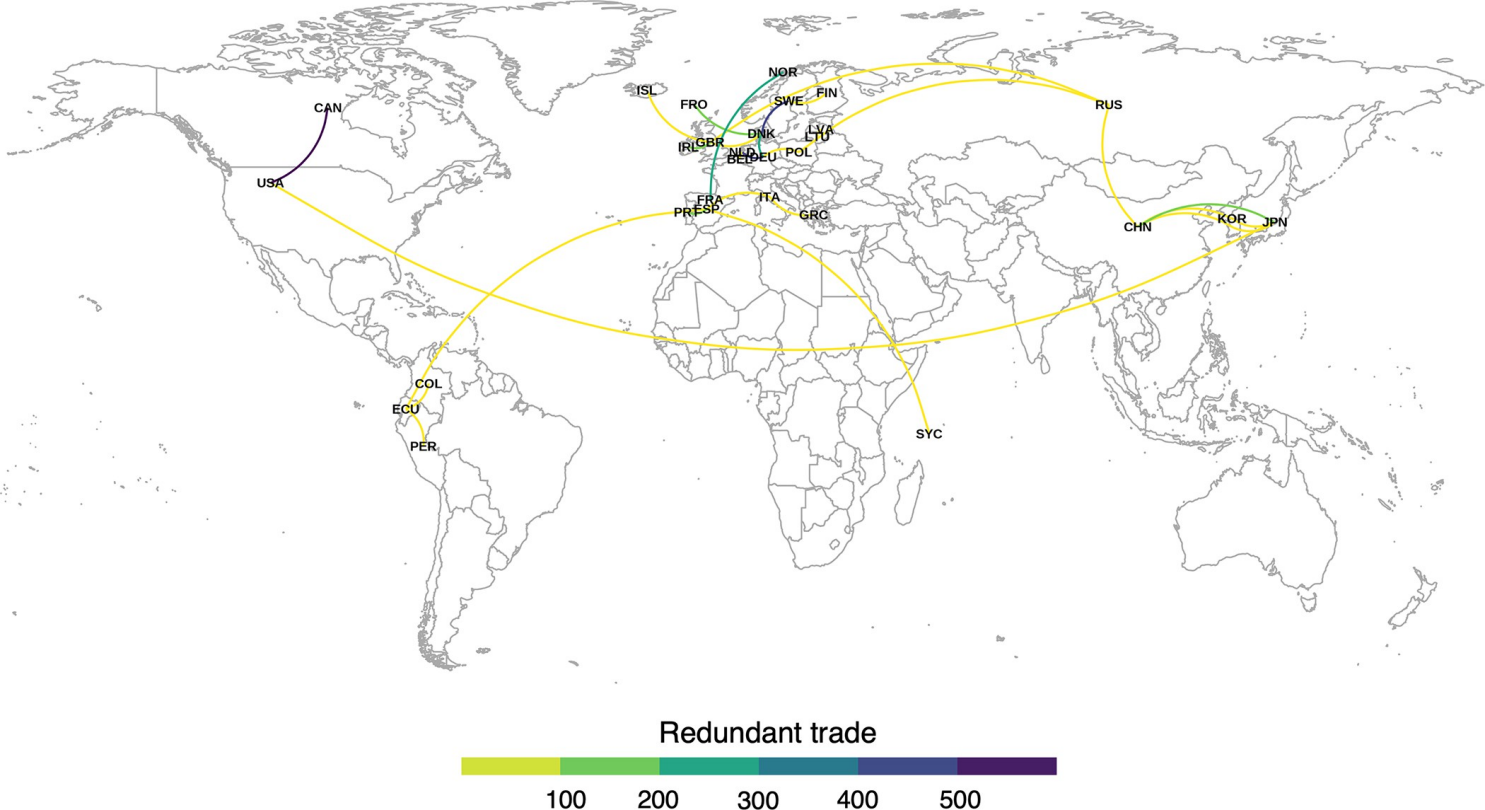
Volume of redundant trade between the top trade partners. The map depicts connections between trade partners that account for 90% of redundant trade between 2000–2015. Map created in R using rworldmaps package (https://cran.r-project.org/web/packages/rworldmap/index.html) and Natural Earth data (http://www.naturalearthdata.com/).

Redundant trade occurs between 274 different trade partners, but the majority (51%) of redundant trade was between five trade partners (Fig 4, see S6 Table for a full list of redundant trade partners): Canada and the United States (15%; 1.15 Mt), Germany and the Netherlands (11.8%; 0.9 Mt); Denmark and Sweden (10.6%; 0.8 Mt); Germany and Denmark (7.1%; 0.5 Mt); and France and Norway (7%; 0.5 Mt). Most trade partners (191, 70%) were within the same continent, while 39% (106) were within the same subregion (e.g., Northern America, Western Europe, etc.).

**Fig 5:**
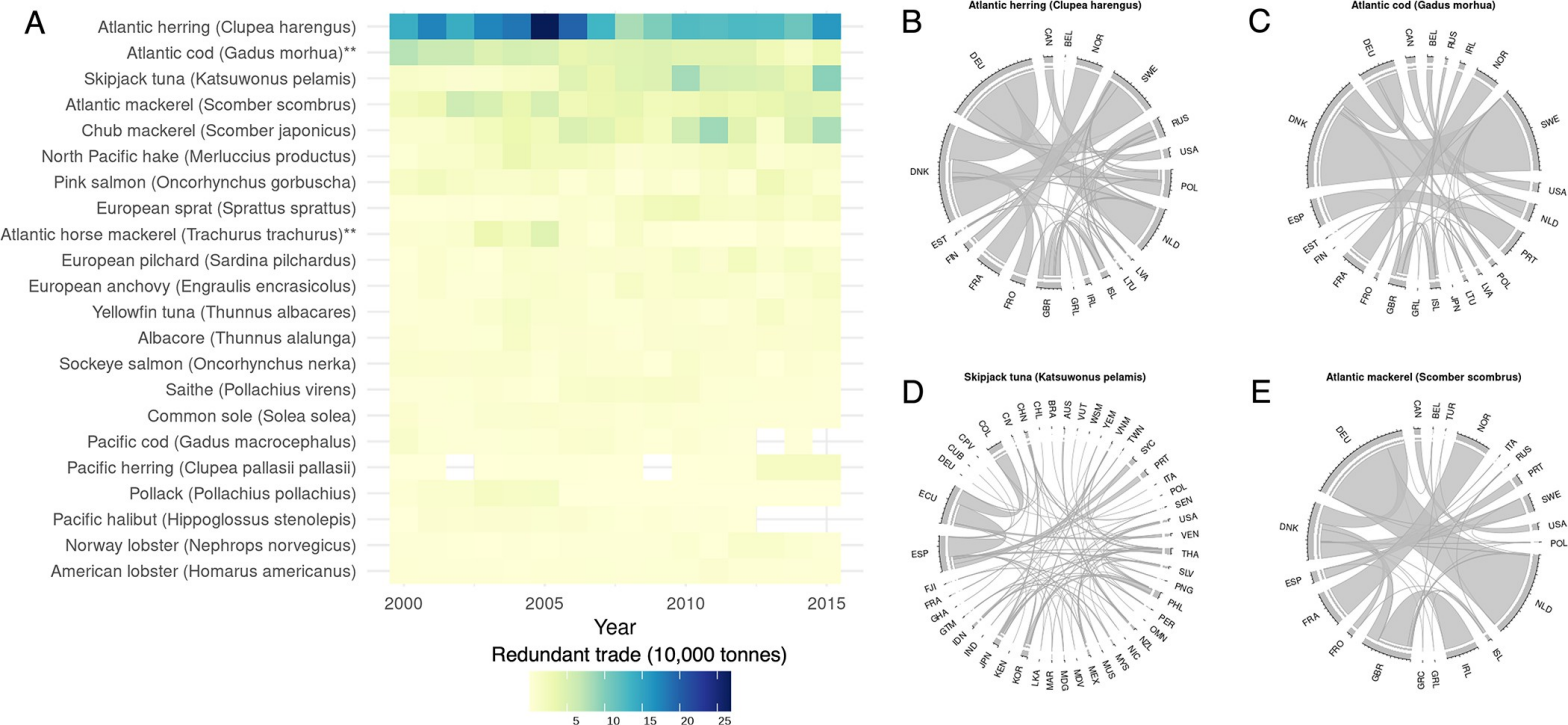
Volume of the most redundantly traded species. The table depicts redundant trade that account for 90% of redundant trade through time (A). Country trade partners for the top four redundantly traded species (~50% of redundant trade) from 2000–2015 including Atlantic herring (B), skipjack tuna (C), Atlantic cod (D) and Atlantic mackerel (E). Thickness of the circle plot connections represents relative tonnes traded (thicker connection equates to greater trade between two countries). The ** symbols denote species that are listed as vulnerable on the IUCN Red List of threatened species.

Between 2000 and 2015, a total of 272 species (identified to the species level) were traded, 114 (~42%) of which were redundantly traded. Spain redundantly traded the greatest number of species (48), followed by Portugal (46), Germany (39), Denmark (37) and the United Kingdom (37), with ~9 redundantly traded species for each country, on average (Fig 3B). Just 22 species made up 90% of redundant trade, with four accounting for 56% (Fig 5). These include Atlantic herring (31%; 2,380.9 thousand tonnes), Atlantic cod (9.4%; 724.5), Skipjack tuna (8%; 619.5), and Atlantic mackerel (7.7%; 519.27). Nearly 50% of Atlantic herring is traded between just four countries (Germany and Netherlands (17.9%; 426.9 thousand tonnes), Denmark and Sweden (16.4%; 389.6), and Germany and Denmark (14.7%, 350.9)). Over 50% of Atlantic cod is traded between Denmark and Sweden (38%; 278.8) and Spain and Portugal (13%; 92.7). The main trade partners for skipjack tuna were Ecuador and Spain (27%; 169.8 thousand tonnes) and Colombia and Ecuador (19%; 120.5). Finally, Atlantic mackerel is mostly traded between Germany and Netherlands (36%; 211.9 thousand tonnes) and France and Norway (14%; 88.9). Of redundantly traded species, two are listed as endangered (Senegalese hake (*Merluccius senegalensis*) and Japanese sea cucumber (*Apostichopus japonicus*)), seven as vulnerable and four as near threatened on the IUCN Red List of threatened species (S7 Table). Endangered and vulnerable species account for 12.7% (1 Mt) of redundant trade by volume. A further 46 species (40% of redundantly traded species) are data deficient or have not been assessed by the IUCN.

## Discussion

We found that the majority of countries participated in redundant two-way trade across the global seafood trade network between 2000 and 2015. There are numerous social (e.g., taste preferences), economic (e.g., profit maximization), political (e.g., trade agreements), and ecological (e.g., resource availability, habitat condition) factors that determine why seafood is traded between two countries [19]. Two-way trade (i.e., exchange of functionally similar goods or commodities between two countries) [20] of seafood is no exception. We acknowledge that our results are dependent upon the quality of trade data, which is impacted by the well-known fact that the traceability of global seafood supply chains is poor [8, 21]. Regardless, we have highlighted an approach to quantify redundant two-way trade in the seafood sector to provide pathways for further research to improve seafood supply chains–a common goal in many national and global resource management policies, including the United Nations 2030 Sustainable Development Goals. Achieving a nature positive future requires evaluation across entire value chains [22]. We identify redundant trade as one potential leverage point to avoid impacts without compromising seafood supply.

Although redundant trade only accounts for a small fraction of global seafood trade (2.6%-4.6%, depending on the year), and potential motivations for participating in redundant trade are complex, there are several potential benefits for reducing its occurrence. Eliminating redundant trade could shorten supply chains by keeping goods within the producing country. This would reduce impact displacement without compromising supply. For example, shortening supply chains could reduce environmental impacts of trade, such as the spread of pests and greenhouse gas emissions (particularly for countries trading across large geographic distances). For intercontinental trade partners, like Ecuador and Spain or China and Russia (Fig 4), retaining their own wild-caught fisheries production could reduce carbon emissions from freight, which currently accounts for 7% of global emissions and is expected to increase 4-fold by 2050 [23, 24]. In the same vein, reducing redundant trade could improve impact accounting by limiting the displacement of environmental and socioeconomic impacts through trade and improving traceability, a pervasive problem in seafood production that is complicated by complex trade networks that would be streamlined if redundant trade was eliminated. Eliminating redundant two-way trade could decrease trade-dependence of certain countries, reducing potential impacts of world trade disruptions, such as during the COVID-19 pandemic [25].

On the other hand, trade can also benefit the environment by increasing use efficiency and reducing waste [2]. There is contradictory evidence that two-way traded agricultural commodities may in fact have positive environmental effects [24, 26], highlighting the need to investigate these patterns specifically for the seafood sector. For example, a study by Farmery et al. (2015) found that carbon footprints of imported and domestic seafood were similar in Australia since the capture or on-farm stage is usually the greatest source of carbon emissions [27].

Our finding that redundant seafood trade has nearly doubled between 2000 and 2015 is consistent with the rise of seafood consumption and trade in general [2, 28].We found that nearly 70% of redundant trade occurs between just 10 countries (Denmark, Germany, the United States, Canada, the Netherlands, Sweden, Spain, the United Kingdom, France, and Norway). The top trade partners participating in redundant two-way trade are close neighbours (Canada-United States, Germany-Netherlands, Denmark-Sweden, Germany-Denmark, France-Norway), most of which are part of the European Union.

Other studies have found large increases in seafood trade within regions, largely driven by regional free trade agreements [2]. Common Fisheries Policy and trade agreements across the European Union and the United States-Mexico-Canada Agreement in North America likely support beneficial trade across these areas, along with strong political and economic bonds, which together may incentivize redundant two-way trade. However, as transportation and communication costs decrease there may be an increase in redundant trade between more distant trade partners [29].

Interestingly, many of the same countries participating in redundant trade have been identified as top countries involved in other unsustainable aspects of the seafood supply chain, including importing and fishing for endangered species [30], importing from and fishing in places that have poorly managed fisheries [13], and subsidising fisheries [31]. This suggests that a stronger focus on environmental sustainability within EU and North American seafood trade policies could set a precedence for global sustainable trade and development.

Our results provide a first estimate of how much redundant seafood trade occurs, but not why or when redundant trade is occurring. In addition, some redundant trade may occur due to the exchange of the same species as different products (e.g., fresh and canned tuna), which could not be accounted for in our analysis. Some redundant trade may be explained by seasonality and temporal fishing management restrictions, such as limiting the catch of a species in different countries at different times. In such cases, countries would rely on imports to meet seafood demands during some months but would be relied upon during other months to achieve other countries seafood demands. Fine scale data regarding processing stages, trade networks across countries and regions, and seasonal variation would shed light on potential efficiencies of such trade and should be considered in future work when available.

Further research is required to uncover why we see redundant trade between certain countries (e.g., Canada-United States; Germany-Netherlands; Denmark-Sweden; Denmark-Germany; France-Norway) or high proportions of seafood trade that is classified as redundant (e.g., Belgium, France, Germany). This will help to understand what contexts are driving this trade, such as geographic location, financial incentives (i.e., profit maximization), processing costs, seasonality, or political motives. Looking to countries that trade relatively large amounts of seafood, with little classified as redundant (e.g., Spain), may also help answer the questions around why redundant seafood trade occurs. Understanding socioeconomic and environmental implications of this behaviour may disincentivize suppliers, which are increasingly encouraged to incorporate nature and socioeconomic considerations in their decision making (e.g., Nature-related financial disclosures).

We found that just four species (Atlantic herring, Atlantic cod, Skipjack tuna and Atlantic mackerel) are responsible for 50% of redundant trade. Two of the most redundantly traded species (Atlantic cod and Atlantic horse mackerel) are listed as Vulnerable on the IUCN Red List of Threatened Species. International agreements such as the Convention on International Trade in Endangered Species of Wild Fauna (CITES) as well as national or region-scale species recovery and fisheries management measures could be used to reduce such practices. However, eliminating redundant trade would not reduce the amount of harvest of these threatened species, which points to previous arguments in the literature suggesting that industrial-scale harvest and trade of species at risk of extinction should not be legal [30].

We note the high variability in trade reporting at the species level between countries, and its likely impact on our results. China for example, is the largest seafood trader globally and fell just outside of the top 10 redundant seafood traders (12^th^). However, China only reports 27% of its trade at the species level, whereas the top 10 countries report an average of 79% of trade to the species level (S5 Table). We excluded nearly half of the trade dataset because it was not reported to the taxonomic level (e.g., ‘miscellaneous marine molluscs’ or ‘marine animals’), which likely led to underreporting or redundant trade. On the other hand, assessing redundant trade by product rather than species may result in lower estimates of redundant trade if products are deemed to be different and thus not redundant. Countries should not be penalised for more accurate levels of reporting, and our results join the calls for improved transparency and reporting in the seafood sector [32, 33].

There are several important aspects of the data and our analysis that need to be considered when interpreting our results. As mentioned previously, aggregation across time, space, and product within the seafood trade dataset likely overestimates our results in some instances and underestimates in others. Quantifying redundant trade at a finer resultion across these scales and further exploring its drivers should be a priority when data becomes available. Further, our analysis is not representative of all fish and fish products as we focused on wild-caught fisheries and did not assess aquaculture trade. Aquaculture is currently responsible for more than half the world’s seafood production, which may provide a different picture than our findings [28]. However, including aquaculture would create complexities for comparability of products and discerning country motivation for participating in redundant trade. Additional datasets should be explored in future analyses, such as the Food and Agricultural Organisation (FAO) trade partner database and the United Nations Comtrade database. However, there are many complexities and difficulties in comparability due to differences in time scales, commodities (i.e., live weight vs product weight vs value), and lack of distinction between production methods (i.e., wild-caught and aquaculture).

Finally, when exploring the role of redundant trade in seafood sustainability, thought should be given to the role of consumer preference on the demand for certain types of seafood [19]. While we’ve assumed species in our trade dataset represent homogenous goods, this may not always be the case, with consumer preference differentiating parts of the fish, preservation methods or cooked methods thus driving redundant seafood trade [27, 34]. For example, Australia, like many other countries, exports high-value seafood products (e.g., premium tuna species, like bluefin, to Japan) and imports lower value products (e.g., canned skipjack tuna from Thailand) [35]. However, we have accounted for as much of this variation as possible within our dataset by: 1) only investigating trade of the same taxonomic species between two counties; 2) omitting re-exported seafood to reduce records of processed (e.g., canned) seafood; 3) only considering wild-caught, not aquaculture produced seafood. Further work is needed to build on our results to better understand the drivers and costs (social, environmental, and economic) of redundant trade and the potential benefits of its reduction.

Globalisation and technological advancements have allowed seafood trade to steadily increase since the 1970s [19, 36]. There are many economic and social benefits of global trade, but also many inefficiencies that can result in poor environmental and social outcomes. We found that redundant trade occurs globally and has been increasing in recent years, despite still making up a relatively small proportion of total seafood trade. Redundant trade could represent an overlooked leverage point to streamline sustainable trade and thus reduce socio-economic and environmental impacts of seafood globally and should be explored in future studies. Understanding and incorporating such imperative information will be pivotal for meeting global sustainable development objectives.

## Supporting information

S1 Table List of species identified to taxonomic species level for this analysis. (DOCX)

S2 Table List of groups identified to a higher taxonomic level that were excluded from the analysis. (DOCX)

S3 Table Annual volume (tonnes) of wild-caught seafood trade through time for all seafood (’Global’), seafood identified to species level (‘Species’), and redundant two-way trade (‘Redundant’) (2000–2015). (DOCX)

S4 Table Total volume and proportion (of total country trade) of redundant two-way wild-caught seafood trade between 2000–2015 by country. (DOCX)

S5 Table Proportion of total trade identified to species for each country. (DOCX)

S6 Table List of country trade partners that participated in redundant species trad between 2000–2015. (DOCX)

S7 Table List of all species two-way traded, total volume of redundant two-way trade (tonnes), and their IUCN ranking (LC = Least Concern, VU = Vulnerable, NT = Near Threatened, EN = Endangered, DD = Data Deficient, NA = Not Assessed). (DOCX)
