# Supplementary material for: Quantifying global redundant fisheries trade to streamline seafood supply chains
Source: PLoS One. 2024 Jul 10;19(7):e0305779. doi: 10.1371/journal.pone.0305779 (PMC11236095; doi:10.1371/journal.pone.0305779)
Supplement: S1 Table — (DOCX) [file pone.0305779.s001.docx]

# **Supplementary Material – Kuempel et al.** Quantifying global redundant fisheries trade to streamline seafood supply chains

**Table S1.** List of species identified to taxonomic species level for this analysis

| **Taxon key** | **Common name** | **Taxon name** |
| --- | --- | --- |
| 601477 | South American pilchard | Sardinops sagax |
| 600367 | Inca scad | Trachurus murphyi |
| 600117 | Chub mackerel | Scomber japonicus |
| 600107 | Skipjack tuna | Katsuwonus pelamis |
| 600324 | South Pacific hake | Merluccius gayi gayi |
| 690078 | Chilean sea urchin | Loxechinus albus |
| 600467 | Patagonian toothfish | Dissostichus eleginoides |
| 690077 | Chilean nylon shrimp | Heterocarpus reedi |
| 600226 | Swordfish | Xiphias gladius |
| 600143 | Yellowfin tuna | Thunnus albacares |
| 600142 | Albacore | Thunnus alalunga |
| 600004 | Anchoveta | Engraulis ringens |
| 690127 | False abalone | Concholepas concholepas |
| 690377 | Southern king crab | Lithodes antarcticus |
| 600322 | Southern hake | Merluccius australis |
| 600334 | Orange roughy | Hoplostethus atlanticus |
| 600320 | Southern blue whiting | Micromesistius australis |
| 600482 | Pink cusk-eel | Genypterus blacodes |
| 690088 | Common octopus | Octopus vulgaris |
| 600066 | European anchovy | Engraulis encrasicolus |
| 600063 | European seabass | Dicentrarchus labrax |
| 600030 | European hake | Merluccius merluccius |
| 601350 | European pilchard | Sardina pilchardus |
| 600481 | Poor cod | Trisopterus minutus |
| 600115 | Atlantic bonito | Sarda sarda |
| 690070 | Caramote prawn | Melicertus kerathurus |
| 601357 | European sprat | Sprattus sprattus |
| 690085 | Common cuttlefish | Sepia officinalis |
| 690109 | Deepwater rose shrimp | Parapenaeus longirostris |
| 600525 | Common sole | Solea solea |
| 600118 | Atlantic mackerel | Scomber scombrus |
| 600147 | Atlantic bluefin tuna | Thunnus thynnus |
| 601756 | Common seabream | Pagrus pagrus |
| 690273 | Norway lobster | Nephrops norvegicus |
| 600141 | West African Spanish mackerel | Scomberomorus tritor |
| 690071 | Caribbean spiny lobster | Panulirus argus |
| 690023 | Argentine shortfin squid | Illex argentinus |
| 601659 | Argentine anchoita | Engraulis anchoita |
| 600325 | Argentine hake | Merluccius hubbsi |
| 690024 | Argentine stiletto shrimp | Artemesia longinaris |
| 690686 | Patagonean scallop | Zygochlamys patagonica |
| 600139 | Piked dogfish | Squalus acanthias |
| 690049 | Blacklip abalone | Haliotis rubra |
| 690696 | Southern rock lobster | Jasus novaehollandiae |
| 600372 | Greenback horse mackerel | Trachurus declivis |
| 690161 | Green rock lobster | Jasus verreauxi |
| 690039 | Australian spiny lobster | Panulirus cygnus |
| 690042 | Banana prawn | Fenneropenaeus merguiensis |
| 600146 | Bigeye tuna | Thunnus obesus |
| 600018 | Nassau grouper | Epinephelus striatus |
| 690163 | Green tiger prawn | Penaeus semisulcatus |
| 601863 | Pink ear emperor | Lethrinus lentjan |
| 600121 | Narrow-barred Spanish mackerel | Scomberomorus commerson |
| 601846 | Spangled emperor | Lethrinus nebulosus |
| 610220 | Giant seacatfish | Arius thalassinus |
| 600089 | Wahoo | Acanthocybium solandri |
| 600078 | Atlantic sailfish | Istiophorus albicans |
| 600006 | Common dolphinfish | Coryphaena hippurus |
| 601381 | Haddock | Melanogrammus aeglefinus |
| 600024 | Atlantic herring | Clupea harengus |
| 600034 | Pollack | Pollachius pollachius |
| 690125 | European lobster | Homarus gammarus |
| 600033 | Ling | Molva molva |
| 600029 | Whiting | Merlangius merlangus |
| 600695 | Dab | Limanda limanda |
| 600069 | Atlantic cod | Gadus morhua |
| 601342 | European plaice | Pleuronectes platessa |
| 600028 | Megrim | Lepidorhombus whiffiagonis |
| 690119 | Edible crab | Cancer pagurus |
| 601371 | Atlantic halibut | Hippoglossus hippoglossus |
| 601365 | Atlantic horse mackerel | Trachurus trachurus |
| 690156 | Great Atlantic scallop | Pecten maximus |
| 601382 | Lemon sole | Microstomus kitt |
| 601343 | Saithe | Pollachius virens |
| 690091 | Common shrimp | Crangon crangon |
| 600845 | Smallspotted catshark | Scyliorhinus canicula |
| 600854 | Nursehound | Scyliorhinus stellaris |
| 690032 | Atlantic seabob | Xiphopenaeus kroyeri |
| 690327 | Redspotted shrimp | Farfantepenaeus brasiliensis |
| 600171 | Southern red snapper | Lutjanus purpureus |
| 601078 | Blackfin goosefish | Lophius gastrophysus |
| 600216 | Atlantic blue marlin | Makaira nigricans |
| 600369 | Rough scad | Trachurus lathami |
| 690241 | Mediterranean mussel | Mytilus galloprovincialis |
| 601278 | Mediterranean horse mackerel | Trachurus mediterraneus |
| 605382 | Cape horse mackerel | Trachurus capensis |
| 600512 | Sablefish | Anoplopoma fimbria |
| 600241 | Chum salmon | Oncorhynchus keta |
| 690115 | Dungeness crab | Cancer magister |
| 690033 | Atlantic surf clam | Spisula solidissima |
| 600504 | Pacific ocean perch | Sebastes alutus |
| 600240 | Pink salmon | Oncorhynchus gorbuscha |
| 600243 | Sockeye salmon | Oncorhynchus nerka |
| 690031 | Atlantic rock crab | Cancer irroratus |
| 600516 | Greenland halibut | Reinhardtius hippoglossoides |
| 690269 | Northern prawn | Pandalus borealis |
| 690010 | American lobster | Homarus americanus |
| 690011 | American sea scallop | Placopecten magellanicus |
| 600514 | Pacific halibut | Hippoglossus stenolepis |
| 600051 | Tusk | Brosme brosme |
| 600521 | Yellowtail flounder | Limanda ferruginea |
| 600326 | North Pacific hake | Merluccius productus |
| 600252 | Capelin | Mallotus villosus |
| 602674 | Broad whitefish | Coregonus nasus |
| 602669 | Arctic cisco | Coregonus autumnalis |
| 690203 | Jonah crab | Cancer borealis |
| 690399 | Stone king crab | Lithodes maia |
| 600245 | Coho salmon | Oncorhynchus kisutch |
| 602501 | Wolf-fish | Anarhichas lupus |
| 600239 | Rainbow trout | Oncorhynchus mykiss |
| 600244 | Chinook salmon | Oncorhynchus tshawytscha |
| 603811 | Spotted wolffish | Anarhichas minor |
| 603810 | Northern wolffish | Anarhichas denticulatus |
| 614063 | Argentine goatfish | Mullus argentinae |
| 690205 | Juan Fernandez rock lobster | Jasus frontalis |
| 600113 | Eastern Pacific bonito | Sarda chiliensis chiliensis |
| 600416 | Yellow croaker | Larimichthys polyactis |
| 601520 | Pacific herring | Clupea pallasii pallasii |
| 601663 | Japanese anchovy | Engraulis japonicus |
| 690379 | Southern rough shrimp | Trachysalambria curvirostris |
| 601288 | Largehead hairtail | Trichiurus lepturus |
| 690133 | Fleshy prawn | Fenneropenaeus chinensis |
| 600298 | Daggertooth pike conger | Muraenesox cinereus |
| 600303 | Pacific saury | Cololabis saira |
| 690198 | Japanese flying squid | Todarodes pacificus |
| 690228 | Longlegged spiny lobster | Panulirus longipes |
| 600218 | Indo-Pacific blue marlin | Makaira mazara |
| 690219 | Kuruma prawn | Marsupenaeus japonicus |
| 600145 | Southern bluefin tuna | Thunnus maccoyii |
| 614290 | Pacific bluefin tuna | Thunnus orientalis |
| 600131 | Japanese Spanish mackerel | Scomberomorus niphonius |
| 600366 | Japanese jack mackerel | Trachurus japonicus |
| 690328 | Redtail prawn | Penaeus penicillatus |
| 690151 | Giant tiger prawn | Penaeus monodon |
| 690288 | Atlantic seabob | Xiphopenaeus kroyeri |
| 600136 | Pacific sierra | Scomberomorus sierra |
| 600098 | Black skipjack | Euthynnus lineatus |
| 690433 | Western white shrimp | Litopenaeus occidentalis |
| 600548 | Pacific anchoveta | Cetengraulis mysticetus |
| 690619 | Blue spiny lobster | Panulirus gracilis |
| 600077 | Indo-Pacific sailfish | Istiophorus platypterus |
| 690100 | Crystal shrimp | Farfantepenaeus brevirostris |
| 600223 | Striped marlin | Tetrapturus audax |
| 603915 | Shortbill spearfish | Tetrapturus angustirostris |
| 600219 | Atlantic white marlin | Tetrapturus albidus |
| 600097 | Little tunny | Euthynnus alletteratus |
| 690268 | Northern pink shrimp | Farfantepenaeus duorarum |
| 600716 | Angler | Lophius piscatorius |
| 690090 | Common prawn | Palaemon serratus |
| 600238 | Sea trout | Salmo trutta trutta |
| 690053 | Blue mussel | Mytilus edulis |
| 600236 | Atlantic salmon | Salmo salar |
| 601383 | Blue ling | Molva dypterygia |
| 600232 | Common whitefish | Coregonus lavaretus |
| 600233 | Houting | Coregonus oxyrinchus |
| 600501 | Ocean perch | Sebastes marinus |
| 600505 | Deepwater redfish | Sebastes mentella |
| 600391 | Atlantic pomfret | Brama brama |
| 690671 | Surf clam | Spisula solida |
| 600031 | Blue whiting | Micromesistius poutassou |
| 600331 | Onion-eye grenadier | Macrourus berglax |
| 690232 | Mangrove cupped oyster | Crassostrea rhizophorae |
| 690283 | Pacific cupped oyster | Crassostrea gigas |
| 690444 | Yellowleg shrimp | Farfantepenaeus californiensis |
| 690054 | Blue shrimp | Litopenaeus stylirostris |
| 690440 | Whiteleg shrimp | Litopenaeus vannamei |
| 600148 | Longtail tuna | Thunnus tonggol |
| 600096 | Kawakawa | Euthynnus affinis |
| 606426 | Squirefish | Pagrus auratus |
| 601827 | Panama hake | Merluccius angustimanus |
| 604988 | Shortspine African angler | Lophius vaillanti |
| 600313 | White hake | Urophycis tenuis |
| 690271 | Northern shortfin squid | Illex illecebrosus |
| 600323 | Silver hake | Merluccius bilinearis |
| 600532 | American angler | Lophius americanus |
| 690059 | Broadtail shortfin squid | Illex coindetii |
| 690680 | Delta prawn | Palaemon longirostris |
| 690631 | Venus clam | Spisula ovalis |
| 690420 | Tuberculate abalone | Haliotis tuberculata |
| 690378 | Southern pink shrimp | Farfantepenaeus notialis |
| 690168 | Guinea shrimp | Parapenaeopsis atlantica |
| 601661 | Cape anchovy | Engraulis capensis |
| 610256 | Cape monk | Lophius vomerinus |
| 600371 | Cunene horse mackerel | Trachurus trecae |
| 600327 | Benguela hake | Merluccius polli |
| 607039 | Antarctic toothfish | Dissostichus mawsoni |
| 690003 | Aesop shrimp | Pandalus montagui |
| 600217 | Black marlin | Makaira indica |
| 690123 | European flat oyster | Ostrea edulis |
| 690404 | Striped venus | Venus (=Chamelea) gallina |
| 600309 | Greenland cod | Gadus ogac |
| 600144 | Blackfin tuna | Thunnus atlanticus |
| 600015 | Red hind | Epinephelus guttatus |
| 600094 | Frigate tuna | Auxis thazard thazard |
| 690166 | Grooved carpet shell | Ruditapes decussatus |
| 600319 | Polar cod | Boreogadus saida |
| 690694 | Whitebelly prawn | Nematopalaemon schmitti |
| 600062 | Lumpsucker | Cyclopterus lumpus |
| 601511 | Indian oil sardine | Sardinella longiceps |
| 600116 | Blue mackerel | Scomber australasicus |
| 604461 | Greasy grouper | Epinephelus tauvina |
| 604923 | Honeycomb grouper | Epinephelus merra |
| 600114 | Striped bonito | Sarda orientalis |
| 690124 | European flying squid | Todarodes sagittatus |
| 600308 | Pacific cod | Gadus macrocephalus |
| 690146 | Giant abalone | Haliotis gigantea |
| 690201 | Japanese sea cucumber | Apostichopus japonicus |
| 600513 | Indian spiny turbot | Psettodes erumei |
| 601352 | Norway redfish | Sebastes viviparus |
| 690180 | Horned turban | Turbo cornutus |
| 600487 | Pacific sandeel | Ammodytes personatus |
| 600510 | Okhostk atka mackerel | Pleurogrammus azonus |
| 690356 | Shiba shrimp | Metapenaeus joyneri |
| 601370 | John dory | Zeus faber |
| 600312 | Red hake | Urophycis chuss |
| 600374 | Indian scad | Decapterus russelli |
| 600426 | White grouper | Epinephelus aeneus |
| 690009 | American cupped oyster | Crassostrea virginica |
| 601664 | Californian anchovy | Engraulis mordax |
| 600405 | Spotted weakfish | Cynoscion nebulosus |
| 600368 | Pacific jack mackerel | Trachurus symmetricus |
| 603542 | Cobia | Rachycentron canadum |
| 601164 | Gilthead seabream | Sparus aurata |
| 690754 | Pod razor | Ensis ensis |
| 690321 | Red rock lobster | Jasus edwardsii |
| 600507 | Bluefin gurnard | Chelidonichthys kumu |
| 600489 | Snoek | Thyrsites atun |
| 612962 | Black oreo | Allocyttus niger |
| 690610 | Northern nylon shrimp | Heterocarpus vicarius |
| 600152 | Spotted rose snapper | Lutjanus guttatus |
| 600346 | Barramundi | Lates calcarifer |
| 614693 | Peruvian hake | Merluccius gayi peruanus |
| 600321 | Senegalese hake | Merluccius senegalensis |
| 600027 | Fourspotted megrim | Lepidorhombus boscii |
| 600311 | Brazilian codling | Urophycis brasiliensis |
| 605094 | Black-bellied angler | Lophius budegassa |
| 690383 | Speckled shrimp | Metapenaeus monoceros |
| 690189 | Indian white prawn | Fenneropenaeus indicus |
| 690470 | Neon flying squid | Ommastrephes bartramii |
| 604540 | Bluespotted seabream | Pagrus caeruleostictus |
| 600093 | Bullet tuna | Auxis rochei rochei |
| 605066 | Redbanded seabream | Pagrus auriga |
| 690313 | Queen crab | Chionoecetes opilio |
| 600399 | Silver grunt | Pomadasys argenteus |
| 600394 | Spotted seabass | Dicentrarchus punctatus |
| 600106 | Dogtooth tuna | Gymnosarda unicolor |
| 600111 | Indian mackerel | Rastrelliger kanagurta |
| 600108 | Plain bonito | Orcynopsis unicolor |
| 600485 | Kingklip | Genypterus capensis |
| 607126 | Smalleye moray cod | Muraenolepis microps |
| 601456 | Whiteheads round herring | Etrumeus whiteheadi |
| 601828 | Shallow-water Cape hake | Merluccius capensis |
| 690087 | Common edible cockle | Cardium edule |
| 690309 | Pullet carpet shell | Tapes pullastra |
| 600220 | Longbill spearfish | Tetrapturus pfluegeri |
| 601279 | Blue jack mackerel | Trachurus picturatus |
| 601466 | Falkland sprat | Sprattus fuegensis |
| 600231 | Vendace | Coregonus albula |
| 690432 | Western king prawn | Melicertus latisulcatus |
| 606465 | Orange-spotted grouper | Epinephelus coioides |
| 600790 | Red mullet | Mullus barbatus barbatus |
| 600893 | Common pandora | Pagellus erythrinus |
| 600044 | East Atlantic red gurnard | Aspitrigla cuculus |
| 607138 | Bigeye grenadier | Macrourus holotrachys |
| 600068 | Grey gurnard | Eutrigla gurnardus |
| 601366 | Tub gurnard | Chelidonichthys lucerna |
| 600520 | Yellowfin sole | Limanda aspera |
| 690272 | Northern white shrimp | Litopenaeus setiferus |
| 690660 | Ocean shrimp | Pandalus jordani |
| 604037 | Atka mackerel | Pleurogrammus monopterygius |
| 601589 | Gulf menhaden | Brevoortia patronus |
| 690265 | Northern brown shrimp | Farfantepenaeus aztecus |
| 600407 | South American striped weakfish | Cynoscion striatus |
| 690338 | Sao Paulo shrimp | Farfantepenaeus paulensis |
| 690341 | Scalloped spiny lobster | Panulirus homarus |
