# Supplementary material for: Quantifying global redundant fisheries trade to streamline seafood supply chains
Source: PLoS One. 2024 Jul 10;19(7):e0305779. doi: 10.1371/journal.pone.0305779 (PMC11236095; doi:10.1371/journal.pone.0305779)
Supplement: S2 Table — (DOCX) [file pone.0305779.s002.docx]

# **Supplementary Material – Kuempel et al.** Quantifying global redundant fisheries trade to streamline seafood supply chains

**Table S2.** List of groups identified to a higher taxonomic level that were excluded from the analysis

| **Taxon key** | **Common name** | **Taxon name** |
| --- | --- | --- |
| 400319 | Pomfrets | Bramidae |
| 490036 | King crabs | Lithodidae |
| 490097 | Marine crabs | Brachyura |
| 100039 | Marine fishes | Marine fishes not identified |
| 300061 | Flatfishes | Pleuronectiformes |
| 390007 | Octopuses | Octopoda |
| 100000 | Marine animals | Marine animals |
| 400517 | Filefishes | Monacanthidae |
| 100058 | Marine molluscs | Miscellaneous marine molluscs |
| 100047 | Marine crustaceans | Miscellaneous marine crustaceans |
| 590137 | Taquilla clams | Mulinia |
| 400066 | Snake eels | Ophichthidae |
| 390002 | Squids | Teuthida |
| 490055 | Scallops | Pectinidae |
| 400331 | Drums or croakers | Sciaenidae |
| 400240 | Alfonsinos | Berycidae |
| 501327 | Western croakers | Micropogonias |
| 400208 | Sauries | Scomberesocidae |
| 300060 | Perch-likes | Perciformes |
| 500327 | Grenadiers | Macrourus |
| 400425 | Butterfishes | Stromateidae |
| 400185 | Grenadiers or rattails | Macrouridae |
| 100044 | Craylets, squat lobsters | King crabs, squat-lobsters |
| 100056 | Clams, cockles, arkshells | Clams, cockles, arkshells |
| 500142 | Mackerels | Scomber |
| 400359 | Mullets | Mugilidae |
| 400013 | Dogfish sharks | Squalidae |
| 400330 | Porgies | Sparidae |
| 400218 | Silversides | Atherinidae |
| 501145 | Pandoras | Pagellus |
| 500661 | Jack and horse mackerels | Trachurus |
| 100038 | Sharks, rays, skates, etc | Sharks, rays, chimaeras |
| 590082 | Spiny lobsters | Palinurus |
| 501163 | Frigate tuna | Auxis |
| 501940 | Sardinella | Sardinella |
| 100345 | Natantian decapods | Shrimps, prawns |
| 400361 | Threadfins | Polynemidae |
| 500809 | Seabreams (Dentex spp) | Dentex |
| 100043 | Lobsters, spiny-rock lobsters | Homaridae/Palinuridae |
| 100036 | Tunas, bonitos, billfishes | Scombroids |
| 502794 | Weakfishes | Cynoscion |
| 100538 | Various sharks | Sharks, rays, chimaeras |
| 490058 | Cockles | Cardiidae |
| 400384 | Crocodile icefishes | Channichthyidae |
| 500048 | American flounders | Paralichthys |
| 400145 | Sea catfishes | Ariidae |
| 590096 | Penaeus shrimps | Penaeus |
| 400416 | Mackerels, tunas, bonitos | Scombridae |
| 300028 | Herrings | Clupeiformes |
| 390006 | Cuttlefishes | Sepiida |
| 300026 | Eels and morays | Anguilliformes |
| 590079 | Lobsterette | Metanephrops |
| 300044 | Cods | Gadiformes |
| 590083 | Tropical spiny lobsters | Panulirus |
| 500487 | Seerfishes | Scomberomorus |
| 490040 | Slipper lobsters | Scyllaridae |
| 200004 | Sharks and rays | Elasmobranchii |
| 290012 | Sea cucumbers | Holothuroidea |
| 100176 | Starfishes | Starfish and other echinoderms |
| 500357 | Snappers | Lutjanus |
| 400327 | Grunts | Haemulidae |
| 490011 | Cuttlefishes | Sepiidae |
| 400324 | Threadfin breams, Whiptail breams | Nemipteridae |
| 400328 | Emperors or scavengers | Lethrinidae |
| 500366 | Groupers | Epinephelus |
| 400314 | Jacks and pompanos | Carangidae |
| 400323 | Snappers | Lutjanidae |
| 400206 | Flyingfishes | Exocoetidae |
| 400419 | Billfishes | Istiophoridae |
| 590013 | Octopuses | Octopus |
| 503124 | Megrims | Lepidorhombus |
| 590088 | Sand shrimps | Crangon |
| 500112 | Monkfishes | Lophius |
| 500107 | Dogfishes | Squalus |
| 501135 | Redfishes | Sebastes |
| 500654 | Hakes | Merluccius |
| 501096 | Lings | Molva |
| 400515 | Turbots | Scophthalmidae |
| 300059 | Scorpionfishes and flatheads | Scorpaeniformes |
| 400581 | Armored searobins or armored gurnards | Peristediidae |
| 400190 | Goosefishes | Lophiidae |
| 500814 | Lesser catsharks | Scyliorhinus |
| 400362 | Wrasses | Labridae |
| 290004 | Clams | Bivalvia |
| 400445 | Triggerfishes | Balistidae |
| 501941 | Herring | Harengula |
| 400454 | Anchovies | Engraulidae |
| 590014 | Cupped oysters | Crassostrea |
| 502790 | Kingcroakers | Menticirrhus |
| 400332 | Goatfishes | Mullidae |
| 400056 | Moray eels | Muraenidae |
| 505469 | Indo-Pacific mackerels | Rastrelliger |
| 400043 | Herrings, shads, sardines, menhadens | Clupeidae |
| 400405 | Gobies | Gobiidae |
| 500705 | Seabreams and porgies | Diplodus |
| 505799 | Southern soles | Austroglossus |
| 490039 | Spiny lobsters | Palinuridae |
| 400273 | Flatheads | Platycephalidae |
| 400289 | Sea basses, groupers, fairy basslets | Serranidae |
| 290002 | Cephalopods | Cephalopoda |
| 400442 | Tonguefishes | Cynoglossidae |
| 490030 | Palaemonid shrimps | Palaemonidae |
| 400264 | Scorpionfishes or rockfishes | Scorpaenidae |
| 505897 | Wolffish | Anarhichas |
| 501410 | Char | Salvelinus |
| 290011 | Sea urchins | Echinoidea |
| 300009 | Bramble, sleeper, dogfish sharks | Squaliformes |
| 400183 | Cods and haddocks | Gadidae |
| 400402 | Sand lances | Ammodytidae |
| 490054 | Sea mussels | Mytilidae |
| 490041 | Sergestid shrimp | Sergestidae |
| 400160 | Lizardfishes | Synodontidae |
| 500136 | Salmon and trout | Salmo |
| 290006 | Gastropods | Gastropoda |
| 501132 | Tropical goatfishes | Upeneus |
| 590091 | Metapenaeus shrimps | Metapenaeus |
| 400308 | Tilefishes | Malacanthidae |
| 400364 | Parrotfishes | Scaridae |
| 400307 | Smelt-whitings | Sillaginidae |
| 400326 | Mojarras | Gerreidae |
| 400441 | Soles | Soleidae |
| 490043 | Penaeid shrimps | Penaeidae |
| 590125 | Ark clams | Arca |
| 590024 | Horned and musky octopuses | Eledone |
| 490060 | Venus clams | Veneridae |
| 509303 | Gunnels, forkbeards | Phycis |
| 501551 | Porgies | Calamus |
| 500467 | Jacks | Caranx |
| 501905 | Tuna | Thunnus |
| 590146 | Surf clam | Spisula |
| 400076 | Salmonids | Salmonidae |
| 590093 | Pandalus shrimps | Pandalus |
| 502724 | Pacific salmon | Oncorhynchus |
| 502682 | European/spotted seabass | Dicentrarchus |
| 100139 | Finfishes | Marine fishes not identified |
| 400252 | Boarfishes | Caproidae |
| 590070 | Lithodes | Lithodes |
| 400207 | Needlefishes | Belonidae |
| 400415 | Cutlassfishes | Trichiuridae |
| 400439 | Lefteye flounders | Bothidae |
| 501418 | Iberian soles | Microchirus |
| 509366 | Antarctic rockcods | Trematomus |
| 500686 | Rocklings | Gaidropsarus |
| 100338 | Cartilaginous fishes | Sharks, rays, chimaeras |
| 400180 | Codlings | Moridae |
| 400266 | Searobins | Triglidae |
| 490124 | Squat lobsters | Galatheidae |
| 400412 | Surgeonfishes, tangs, unicornfishes | Acanthuridae |
| 400304 | Cardinalfishes | Apogonidae |
| 490004 | Arrow squids | Ommastrephidae |
| 300008 | Ground sharks | Carcharhiniformes |
| 500352 | Shads | Alosa |
| 590112 | Flat oysters | Ostrea |
| 490044 | Crongonid shrimp | Crangonidae |
| 490012 | Octopuses | Octopodidae |
| 590117 | Mussels | Mytilus |
| 500640 | Catsharks | Galeus |
| 500928 | Amberjacks | Seriola |
| 490046 | Norther shrimps | Pandalidae |
| 590073 | King crabs | Paralithodes |
| 490052 | Mantis shrimps | Squillidae |
| 490042 | Aristeid shrimps | Aristeidae |
| 500903 | Seabreams (Pagrus spp) | Pagrus |
| 400440 | Righteye flounders | Pleuronectidae |
| 400010 | Cat sharks | Scyliorhinidae |
| 502102 | Scads | Decapterus |
| 490063 | Mactra surf clams | Mactridae |
| 400318 | Slimys, slipmouths, or ponyfishes | Leiognathidae |
| 507720 | Indo-Pacific anchovies | Stolephorus |
| 400459 | Fusiliers | Caesionidae |
| 400377 | Weeverfishes | Trachinidae |
| 490061 | Razor clams, knife clams | Solenidae |
| 490031 | Swimming crabs | Portunidae |
| 500618 | Soles | Solea |
| 400084 | Deep-sea smelts | Bathylagidae |
| 400448 | Puffers | Tetraodontidae |
| 400421 | Medusafishes | Centrolophidae |
| 506253 | Whitefishes | Coregonus |
| 502417 | Cusk eels | Genypterus |
| 500468 | Queenfishes | Scomberoides |
| 503152 | Croakers | Pseudotolithus |
| 400479 | Bonnetmouths | Inermiidae |
| 400313 | Remoras | Echeneidae |
| 590046 | Abalones | Haliotis |
| 504872 | Toothfishes | Dissostichus |
| 500598 | Pompanos | Trachinotus |
| 590145 | Razor clams | Solen |
| 590051 | Stromboid conchs | Strombus |
| 400356 | Trumpeters | Latridae |
| 400382 | Cod icefishes | Nototheniidae |
| 504140 | Frostfishes | Benthodesmus |
| 400249 | Dories | Zeidae |
| 400250 | Oreos | Oreosomatidae |
| 400475 | Halfbeaks | Hemiramphidae |
| 590116 | Horse mussels | Modiolus |
| 590094 | Shrimps | Parapenaeopsis |
| 590010 | Common squids | Loligo |
| 590124 | Anadara clams | Anadara |
| 400414 | Snake mackerels | Gempylidae |
| 100339 | Pelagic fishes | Marine fishes not identified |
| 590260 | Sculptured shrimps | Sclerocrangon |
| 590074 | Paralomis | Paralomis |
| 590050 | Murex shells | Murex |
| 501550 | Steenbras | Lithognathus |
| 100025 | Diadromous fishes | Miscellaneous diadromous fishes |
| 390017 | Mantis shrimps | Stomatopoda |
| 590111 | Sea snails | Rapana |
| 400011 | Requiem sharks | Carcharhinidae |
| 400062 | Conger and garden eels | Congridae |
| 400340 | Spadefishes, batfishes and scats | Ephippidae |
| 590269 | Gaper clams | Tresus |
