# Supplementary material for: Quantifying global redundant fisheries trade to streamline seafood supply chains
Source: PLoS One. 2024 Jul 10;19(7):e0305779. doi: 10.1371/journal.pone.0305779 (PMC11236095; doi:10.1371/journal.pone.0305779)
Supplement: S3 Table — (DOCX) [file pone.0305779.s003.docx]

# **Supplementary Material – Kuempel et al.** Quantifying global redundant fisheries trade to streamline seafood supply chains

**Table S3.** Annual volume (tonnes) of wild-caught seafood trade through time for all seafood ('Total’), seafood identified to species level ('Species’), and redundant two-way trade (‘Redundant’) (2000-2015)

| **Year** | **Total** | **Species** | **Redundant** |
| --- | --- | --- | --- |
| 2000 | 12131858.1 | 6630785.17 | 392372.546 |
| 2001 | 12665604.4 | 6805496.47 | 436894.838 |
| 2002 | 12732627.7 | 6925223.48 | 427359.597 |
| 2003 | 13001359.8 | 6840037.61 | 490173.393 |
| 2004 | 13894789 | 7325134.81 | 555958.247 |
| 2005 | 14035143.1 | 7258522.36 | 641833.055 |
| 2006 | 15172967.5 | 7967072.11 | 539861.38 |
| 2007 | 15265393.8 | 8023649.91 | 469363.834 |
| 2008 | 15310493.7 | 7809088.09 | 391969.339 |
| 2009 | 15010232.9 | 7733226.9 | 414912.925 |
| 2010 | 16558217.9 | 8261214.71 | 523464.746 |
| 2011 | 17087670.2 | 8436137.8 | 506668.156 |
| 2012 | 16061376.4 | 8515699.89 | 462213.024 |
| 2013 | 16228993.3 | 8417596.43 | 453553.192 |
| 2014 | 16785777 | 8703929.98 | 429277.531 |
| 2015 | 15639630.5 | 8185362.1 | 562617.326 |
