# Supplementary material for: Quantifying global redundant fisheries trade to streamline seafood supply chains
Source: PLoS One. 2024 Jul 10;19(7):e0305779. doi: 10.1371/journal.pone.0305779 (PMC11236095; doi:10.1371/journal.pone.0305779)
Supplement: S4 Table — (DOCX) [file pone.0305779.s004.docx]

# **Supplementary Material – Kuempel et al.** Quantifying global redundant fisheries trade to streamline seafood supply chains

**Table S4.** Total volume and proportion (of total country trade) of redundant two-way wild-caught seafood trade between 2000-2015 by country

| **Country** | **ISO3** | **Total redundant trade (tonnes)** | **Total country trade (tonnes)** | **Proportion of total trade that is redundant** |
| --- | --- | --- | --- | --- |
| Angola | AGO | 13.8000002 | 29243.2222 | 0.00047 |
| Albania | ALB | 344.920997 | 14482.1427 | 0.02382 |
| United Arab Emirates | ARE | 16.8106668 | 92614.3775 | 0.00018 |
| Argentina | ARG | 13040.648 | 4392054.88 | 0.00297 |
| Australia | AUS | 10635.146 | 574547.97 | 0.01851 |
| Belgium | BEL | 57931.4305 | 135679.542 | 0.42697 |
| Bulgaria | BGR | 203.435479 | 16647.3632 | 0.01222 |
| Bahrain | BHR | 840.644666 | 43646.2505 | 0.01926 |
| Bahamas | BHS | 124.291333 | 30773.1188 | 0.00404 |
| Bermuda | BMU | 2.0000001 | 333.374398 | 0.00600 |
| Brazil | BRA | 4880.68397 | 2032369.82 | 0.00240 |
| Barbados | BRB | 12.3074005 | 3223.40908 | 0.00382 |
| Canada | CAN | 577465.523 | 5354284.72 | 0.10785 |
| Chile | CHL | 13054.6968 | 4157011.32 | 0.00314 |
| China | CHN | 252591.86 | 27613344.3 | 0.00915 |
| Côte d'Ivoire | CIV | 6432.20204 | 676637.273 | 0.00951 |
| Congo - Brazzaville | COG | 31.4339987 | 4422.75017 | 0.00711 |
| Cook Islands | COK | 0.89999998 | 8291.45996 | 0.00011 |
| Colombia | COL | 76276.9176 | 923208.725 | 0.08262 |
| Cape Verde | CPV | 37.2066671 | 162200.054 | 0.00023 |
| Costa Rica | CRI | 24.3399996 | 231786.706 | 0.00011 |
| Cuba | CUB | 87.7999988 | 312337.308 | 0.00028 |
| Cyprus | CYP | 50.2003675 | 30265.2343 | 0.00166 |
| Germany | DEU | 831928.253 | 3666861.3 | 0.22688 |
| Denmark | DNK | 877904.812 | 5615015.47 | 0.15635 |
| Dominican Republic | DOM | 0.2 | 28851.5186 | 0.00001 |
| Algeria | DZA | 71.5067335 | 24736.8941 | 0.00289 |
| Ecuador | ECU | 199919.488 | 3082052.28 | 0.06487 |
| Spain | ESP | 405364.492 | 10552033.6 | 0.03842 |
| Estonia | EST | 20085.0683 | 769803.873 | 0.02609 |
| Finland | FIN | 59928.914 | 485464.664 | 0.12345 |
| Fiji | FJI | 4556.95336 | 364440.77 | 0.01250 |
| France | FRA | 284443.1 | 989927.516 | 0.28734 |
| Faroe Islands | FRO | 107671.92 | 1691461.16 | 0.06366 |
| Micronesia (Federated States of) | FSM | 89.7000003 | 267384.916 | 0.00034 |
| United Kingdom | GBR | 290833.172 | 6582957.62 | 0.04418 |
| Georgia | GEO | 17.3000002 | 78883.4145 | 0.00022 |
| Ghana | GHA | 552.699996 | 457758.859 | 0.00121 |
| Greece | GRC | 25585.6058 | 481324.762 | 0.05316 |
| Grenada | GRD | 14.5607 | 4837.34795 | 0.00301 |
| Greenland | GRL | 1145.64074 | 1745256.43 | 0.00066 |
| Guatemala | GTM | 3188.59935 | 186651.27 | 0.01708 |
| Guyana | GUY | 0.1 | 375582.684 | 0.00000 |
| Honduras | HND | 43.4000011 | 42579.3146 | 0.00102 |
| Croatia | HRV | 5017.25382 | 323395.365 | 0.01551 |
| Indonesia | IDN | 11299.4 | 10089672.2 | 0.00112 |
| India | IND | 232.099997 | 7477114.03 | 0.00003 |
| Ireland | IRL | 139593.449 | 2193357.12 | 0.06364 |
| Iceland | ISL | 67780.3713 | 8481822.11 | 0.00799 |
| Italy | ITA | 96394.0549 | 1603751.93 | 0.06011 |
| Japan | JPN | 254185.167 | 5854164.25 | 0.04342 |
| Kenya | KEN | 10.4000004 | 88655.3618 | 0.00012 |
| St. Kitts & Nevis | KNA | 1.40000006 | 10372.8087 | 0.00013 |
| South Korea | KOR | 178075.234 | 7112936.37 | 0.02504 |
| Sri Lanka | LKA | 116.499998 | 239078.442 | 0.00049 |
| Lithuania | LTU | 38948.8725 | 417910.207 | 0.09320 |
| Latvia | LVA | 39990.2834 | 819619.759 | 0.04879 |
| Morocco | MAR | 5353.183 | 5764036.6 | 0.00093 |
| Madagascar | MDG | 2402.09332 | 334838.989 | 0.00717 |
| Maldives | MDV | 70.0000003 | 859244.599 | 0.00008 |
| Mexico | MEX | 19351.0333 | 2016378.97 | 0.00960 |
| Malta | MLT | 2.70000005 | 9688.65186 | 0.00028 |
| Mauritania | MRT | 35.7999998 | 910131.869 | 0.00004 |
| Mauritius | MUS | 9355.61068 | 209963.513 | 0.04456 |
| Malaysia | MYS | 7447.69999 | 2704771.29 | 0.00275 |
| Namibia | NAM | 665.239568 | 6880.49176 | 0.09668 |
| Nicaragua | NIC | 25.4793337 | 128393.087 | 0.00020 |
| Netherlands | NLD | 549292.82 | 4253306.99 | 0.12914 |
| Norway | NOR | 270242.4 | 2124981.98 | 0.12717 |
| New Zealand | NZL | 16063.4327 | 1436687.99 | 0.01118 |
| Oman | OMN | 430.870004 | 525169.857 | 0.00082 |
| Pakistan | PAK | 3.7 | 971653.818 | 0.00000 |
| Panama | PAN | 80.4826667 | 946420.044 | 0.00009 |
| Peru | PER | 38776.5049 | 5519542.63 | 0.00703 |
| Philippines | PHL | 34432.9804 | 2641385.26 | 0.01304 |
| Papua New Guinea | PNG | 2178.16002 | 584701.468 | 0.00373 |
| Poland | POL | 172217.526 | 3641414.33 | 0.04729 |
| Portugal | PRT | 187486.825 | 1980648.1 | 0.09466 |
| French Polynesia | PYF | 7.5 | 20043.6751 | 0.00037 |
| Romania | ROU | 336.553673 | 23943.8288 | 0.01406 |
| Russia | RUS | 152575.428 | 10139626.7 | 0.01505 |
| Saudi Arabia | SAU | 841.378 | 139587.229 | 0.00603 |
| Senegal | SEN | 2816.71904 | 1026041.65 | 0.00275 |
| Singapore | SGP | 0.80000001 | 44137.328 | 0.00002 |
| Solomon Islands | SLB | 4.46000001 | 279546.984 | 0.00002 |
| El Salvador | SLV | 8773.51997 | 244786.557 | 0.03584 |
| Serbia | SRB | 0.84670004 | 403.390108 | 0.00210 |
| São Tomé and Príncipe | STP | 0.1 | 575.329801 | 0.00017 |
| Suriname | SUR | 24.3886669 | 186129 | 0.00013 |
| Slovenia | SVN | 555.087497 | 4579.74266 | 0.12120 |
| Sweden | SWE | 501040.446 | 3043661.51 | 0.16462 |
| Seychelles | SYC | 34594.6591 | 1263355.4 | 0.02738 |
| Turks & Caicos Islands | TCA | 59.199999 | 828.229002 | 0.07148 |
| Togo | TGO | 7.30000007 | 32807.048 | 0.00022 |
| Thailand | THA | 38269.9 | 18927521.7 | 0.00202 |
| Tonga | TON | 4.00000005 | 18185.9234 | 0.00022 |
| Trinidad & Tobago | TTO | 35.1920003 | 64518.9561 | 0.00055 |
| Tunisia | TUN | 5381.28669 | 250399.618 | 0.02149 |
| Turkey | TUR | 7041.04904 | 391689.77 | 0.01798 |
| Taiwan | TWN | 14200.6999 | 1533832.33 | 0.00926 |
| Tanzania | TZA | 0.30000001 | 125853.084 | 0.00000 |
| Ukraine | UKR | 44.6000013 | 1100182.72 | 0.00004 |
| Uruguay | URY | 8413.47668 | 447555.201 | 0.01880 |
| United States | USA | 625935.336 | 10166888.4 | 0.06157 |
| St. Vincent & Grenadines | VCT | 1.95003374 | 22621.8462 | 0.00009 |
| Venezuela | VEN | 13327.2476 | 304412.693 | 0.04378 |
| Vietnam | VNM | 7955.59997 | 11602573.5 | 0.00069 |
| Vanuatu | VUT | 192.700001 | 560522.059 | 0.00034 |
| Samoa | WSM | 30.5333333 | 38660.0327 | 0.00079 |
| Yemen | YEM | 341.819332 | 455105.939 | 0.00075 |
| South Africa | ZAF | 665.239568 | 227570.968 | 0.00292 |
