# Supplementary material for: Quantifying global redundant fisheries trade to streamline seafood supply chains
Source: PLoS One. 2024 Jul 10;19(7):e0305779. doi: 10.1371/journal.pone.0305779 (PMC11236095; doi:10.1371/journal.pone.0305779)
Supplement: S5 Table — (DOCX) [file pone.0305779.s005.docx]

# **Supplementary Material – Kuempel et al.** Quantifying global redundant fisheries trade to streamline seafood supply chains

**Table S5.** Proportion of total trade identified to species for each country

| **Country** | **ISO3** | **Total trade** | **Total trade identified to species** | **Prop. total trade identified to species** |
| --- | --- | --- | --- | --- |
| Aruba | ABW | 102.453 | 0.000 | 0.000 |
| Angola | AGO | 29243.222 | 10059.274 | 0.344 |
| Albania | ALB | 14482.143 | 2431.502 | 0.168 |
| Netherlands Antilles | ANT | 51456.974 | 49013.556 | 0.953 |
| United Arab Emirates | ARE | 92614.377 | 1316.644 | 0.014 |
| Argentina | ARG | 4392054.877 | 3992164.660 | 0.909 |
| Antigua Barb | ATG | 977.473 | 2.000 | 0.002 |
| Australia | AUS | 574547.970 | 235475.449 | 0.410 |
| Belgium | BEL | 135679.542 | 106268.209 | 0.783 |
| Benin | BEN | 6405.428 | 0.000 | 0.000 |
| Bangladesh | BGD | 453779.666 | 0.000 | 0.000 |
| Bulgaria | BGR | 16647.363 | 6851.476 | 0.412 |
| Bahrain | BHR | 43646.251 | 14382.563 | 0.330 |
| Bahamas | BHS | 30773.119 | 29485.074 | 0.958 |
| Bosnia | BIH | 156.824 | 0.000 | 0.000 |
| Belize | BLZ | 8813.818 | 1005.750 | 0.114 |
| Bermuda | BMU | 333.374 | 2.200 | 0.007 |
| Brazil | BRA | 2032369.820 | 422986.662 | 0.208 |
| Barbados | BRB | 3223.409 | 2009.244 | 0.623 |
| Brunei | BRN | 7142.767 | 266.169 | 0.037 |
| Canada | CAN | 5354284.720 | 4861988.125 | 0.908 |
| Chile | CHL | 4157011.322 | 3774091.860 | 0.908 |
| China Main | CHN | 27613344.294 | 7540777.355 | 0.273 |
| Cote d'Ivoire | CIV | 676637.273 | 59391.335 | 0.088 |
| Cameroon | CMR | 28870.503 | 0.000 | 0.000 |
| Congo Dem Rep | COD | 904.833 | 0.000 | 0.000 |
| Congo Rep | COG | 4422.750 | 169.610 | 0.038 |
| Cook Is. | COK | 8291.460 | 804.840 | 0.097 |
| Colombia | COL | 923208.725 | 671343.981 | 0.727 |
| Comoros | COM | 3.540 | 0.000 | 0.000 |
| Cape Verde | CPV | 162200.054 | 110739.278 | 0.683 |
| Costa Rica | CRI | 231786.706 | 46547.480 | 0.201 |
| Cuba | CUB | 312337.308 | 54990.287 | 0.176 |
| Cayman Is | CYM | 502.200 | 0.000 | 0.000 |
| Cyprus | CYP | 30265.234 | 3257.821 | 0.108 |
| Germany | DEU | 3666861.298 | 2603522.811 | 0.710 |
| Djibouti | DJI | 91.367 | 0.000 | 0.000 |
| Dominica | DMA | 11.907 | 7.940 | 0.667 |
| Denmark | DNK | 5615015.467 | 3615027.350 | 0.644 |
| Dominican Republic | DOM | 28851.519 | 409.623 | 0.014 |
| Algeria | DZA | 24736.894 | 8838.079 | 0.357 |
| Ecuador | ECU | 3082052.285 | 2330795.612 | 0.756 |
| Egypt | EGY | 518846.450 | 1192.702 | 0.002 |
| Eritrea | ERI | 958.130 | 0.000 | 0.000 |
| Spain | ESP | 10552033.631 | 6950468.850 | 0.659 |
| Estonia | EST | 769803.873 | 765809.510 | 0.995 |
| Finland | FIN | 485464.664 | 478246.378 | 0.985 |
| Fiji | FJI | 364440.770 | 138299.646 | 0.379 |
| Falkland Is | FLK | 48473.100 | 36552.700 | 0.754 |
| France | FRA | 989927.516 | 683842.535 | 0.691 |
| Faeroe Is | FRO | 1691461.159 | 1638663.137 | 0.969 |
| Micronesia | FSM | 267384.916 | 258823.691 | 0.968 |
| Gabon | GAB | 36635.168 | 331.567 | 0.009 |
| UK | GBR | 6582957.615 | 6123993.718 | 0.930 |
| Georgia | GEO | 78883.414 | 45392.697 | 0.575 |
| Ghana | GHA | 457758.859 | 360614.504 | 0.788 |
| Guinea | GIN | 104790.475 | 6710.179 | 0.064 |
| Gambia | GMB | 19117.299 | 1296.687 | 0.068 |
| Guinea Bissau | GNB | 197813.241 | 1836.200 | 0.009 |
| Eq Guinea | GNQ | 811.600 | 0.000 | 0.000 |
| Greece | GRC | 481324.762 | 171564.929 | 0.356 |
| Grenada | GRD | 4837.348 | 4209.189 | 0.870 |
| Greenland | GRL | 1745256.426 | 1735895.389 | 0.995 |
| Guatemala | GTM | 186651.270 | 155871.916 | 0.835 |
| Guyana | GUY | 375582.684 | 203423.497 | 0.542 |
| Hong Kong | HKG | 649471.111 | 0.000 | 0.000 |
| Honduras | HND | 42579.315 | 2139.000 | 0.050 |
| Croatia | HRV | 323395.365 | 268992.161 | 0.832 |
| Haiti | HTI | 5160.594 | 2290.200 | 0.444 |
| Indonesia | IDN | 10089672.178 | 3459392.198 | 0.343 |
| Isle of Man | IMN | 69.378 | 0.000 | 0.000 |
| India | IND | 7477114.032 | 1925805.863 | 0.258 |
| Ireland | IRL | 2193357.125 | 2038057.752 | 0.929 |
| Iran | IRN | 273591.177 | 14276.146 | 0.052 |
| Iraq | IRQ | 1453.800 | 0.000 | 0.000 |
| Iceland | ISL | 8481822.105 | 8403903.629 | 0.991 |
| Israel | ISR | 8550.963 | 1096.039 | 0.128 |
| Italy | ITA | 1603751.933 | 745811.730 | 0.465 |
| Jamaica | JAM | 5235.986 | 19.920 | 0.004 |
| Channel Is | JEM | 1717.708 | 0.000 | 0.000 |
| Jordan | JOR | 258.465 | 40.370 | 0.156 |
| Japan | JPN | 5854164.246 | 2871053.730 | 0.490 |
| Kenya | KEN | 88655.362 | 450.639 | 0.005 |
| Cambodia | KHM | 370450.903 | 0.000 | 0.000 |
| Kiribati | KIR | 451939.225 | 447116.137 | 0.989 |
| St Kitts Nev | KNA | 10372.809 | 4.527 | 0.000 |
| Korea Rep | KOR | 7112936.368 | 4835838.003 | 0.680 |
| Kuwait | KWT | 5844.783 | 0.000 | 0.000 |
| Lebanon | LBN | 5270.104 | 11.907 | 0.002 |
| Liberia | LBR | 1221.100 | 50.200 | 0.041 |
| Libya | LBY | 15351.714 | 209.047 | 0.014 |
| St Lucia | LCA | 27.607 | 7.160 | 0.259 |
| Sri Lanka | LKA | 239078.442 | 94362.318 | 0.395 |
| Lithuania | LTU | 417910.207 | 319826.943 | 0.765 |
| Latvia | LVA | 819619.759 | 546269.614 | 0.666 |
| Macau | MAC | 5767.390 | 0.000 | 0.000 |
| Morocco | MAR | 5764036.596 | 3695016.189 | 0.641 |
| Madagascar | MDG | 334838.989 | 17896.101 | 0.053 |
| Maldives | MDV | 859244.599 | 845034.749 | 0.983 |
| Mexico | MEX | 2016378.968 | 1248712.649 | 0.619 |
| Marshall Is | MHL | 91041.778 | 79403.400 | 0.872 |
| Malta | MLT | 9688.652 | 3608.485 | 0.372 |
| Myanmar | MMR | 3626764.975 | 0.000 | 0.000 |
| Montenegro | MNE | 960.130 | 21.800 | 0.023 |
| Mozambique | MOZ | 130924.811 | 0.000 | 0.000 |
| Mauritania | MRT | 910131.869 | 128854.747 | 0.142 |
| Saint-Pierre Martinique | MTQ | 8915.269 | 8208.979 | 0.921 |
| Mauritius | MUS | 209963.513 | 55831.681 | 0.266 |
| Malaysia | MYS | 2704771.293 | 77236.256 | 0.029 |
| Mayotte | MYT | 1320.587 | 0.000 | 0.000 |
| Namibia | NAM | 6880.492 | 4703.512 | 0.684 |
| New Caledonia | NCL | 16062.491 | 11600.910 | 0.722 |
| Nigeria | NGA | 276133.655 | 141019.778 | 0.511 |
| Nicaragua | NIC | 128393.087 | 21431.487 | 0.167 |
| Netherlands | NLD | 4253306.992 | 3867242.603 | 0.909 |
| Norway | NOR | 2124981.982 | 1514354.381 | 0.713 |
| Nauru | NRU | 15.355 | 0.000 | 0.000 |
| New Zealand | NZL | 1436687.987 | 802529.953 | 0.559 |
| Oman | OMN | 525169.857 | 13714.769 | 0.026 |
| Pakistan | PAK | 971653.818 | 31903.697 | 0.033 |
| Panama | PAN | 946420.044 | 306028.850 | 0.323 |
| Peru | PER | 5519542.629 | 4403781.543 | 0.798 |
| Philippines | PHL | 2641385.259 | 1555286.276 | 0.589 |
| Palau | PLW | 1913.729 | 14.400 | 0.008 |
| Papua New Guinea | PNG | 584701.468 | 549363.541 | 0.940 |
| Poland | POL | 3641414.331 | 1283413.010 | 0.352 |
| Korea D P Rp | PRK | 680497.326 | 0.000 | 0.000 |
| Portugal | PRT | 1980648.096 | 1170456.987 | 0.591 |
| Palestine | PSE | 509.813 | 0.200 | 0.000 |
| French Polynesia | PYF | 20043.675 | 12074.600 | 0.602 |
| Qatar | QAT | 24360.989 | 0.000 | 0.000 |
| Romania | ROU | 23943.829 | 2555.962 | 0.107 |
| Russia | RUS | 10139626.711 | 7792681.690 | 0.769 |
| Saudi Arabia | SAU | 139587.229 | 44650.690 | 0.320 |
| Sudan | SDN | 2661.955 | 0.000 | 0.000 |
| Senegal | SEN | 1026041.648 | 159202.731 | 0.155 |
| Singapore | SGP | 44137.328 | 18.216 | 0.000 |
| St Helena | SHN | 2028.300 | 576.600 | 0.284 |
| Solomon Is. | SLB | 279546.984 | 236806.793 | 0.847 |
| Sierra Leone | SLE | 85245.944 | 290.360 | 0.003 |
| El Salvador | SLV | 244786.557 | 191795.246 | 0.784 |
| Somalia | SOM | 26657.400 | 0.000 | 0.000 |
| Serbia | SRB | 403.390 | 30.170 | 0.075 |
| Sao Tome Prn | STP | 575.330 | 6.000 | 0.010 |
| Suriname | SUR | 186129.000 | 52443.815 | 0.282 |
| Slovenia | SVN | 4579.743 | 3958.195 | 0.864 |
| Sweden | SWE | 3043661.511 | 2518094.489 | 0.827 |
| Seychelles | SYC | 1263355.401 | 1195359.313 | 0.946 |
| Syria | SYR | 1066.967 | 6.447 | 0.006 |
| Turks Caicos | TCA | 828.229 | 731.529 | 0.883 |
| Togo | TGO | 32807.048 | 2349.169 | 0.072 |
| Thailand | THA | 18927521.705 | 1188548.534 | 0.063 |
| Timor Leste | TLS | 62.830 | 0.663 | 0.011 |
| Tonga | TON | 18185.923 | 426.670 | 0.023 |
| Trinidad Tob | TTO | 64518.956 | 8402.286 | 0.130 |
| Tunisia | TUN | 250399.618 | 178761.846 | 0.714 |
| Turkey | TUR | 391689.770 | 143531.397 | 0.366 |
| Tuvalu | TUV | 13433.969 | 13045.629 | 0.971 |
| Taiwan | TWN | 1533832.330 | 828522.112 | 0.540 |
| Tanzania | TZA | 125853.084 | 667.740 | 0.005 |
| Ukraine | UKR | 1100182.725 | 18295.801 | 0.017 |
| Uruguay | URY | 447555.201 | 393449.379 | 0.879 |
| USA | USA | 10166888.357 | 9308600.846 | 0.916 |
| St Vincent | VCT | 22621.846 | 134.967 | 0.006 |
| Venezuela | VEN | 304412.693 | 147377.563 | 0.484 |
| Viet Nam | VNM | 11602573.522 | 615590.840 | 0.053 |
| Vanuatu | VUT | 560522.059 | 550904.096 | 0.983 |
| Samoa | WSM | 38660.033 | 22522.206 | 0.583 |
| Yemen | YEM | 455105.939 | 39119.401 | 0.086 |
| South Africa | ZAF | 227570.968 | 33484.218 | 0.147 |
