# Supplementary material for: Quantifying global redundant fisheries trade to streamline seafood supply chains
Source: PLoS One. 2024 Jul 10;19(7):e0305779. doi: 10.1371/journal.pone.0305779 (PMC11236095; doi:10.1371/journal.pone.0305779)
Supplement: S6 Table — (DOCX) [file pone.0305779.s006.docx]

# **Supplementary Material – Kuempel et al.** Quantifying global redundant fisheries trade to streamline seafood supply chains

**Table S6.** List of country trade partners that participated in redundant species trad between 2000-2015.

| Trade Partner 1 | Trade Partner 2 | Redundant trade (tonnes) |
| --- | --- | --- |
| AGO | PRT | 27.600 |
| ALB | GRC | 209.236 |
| ALB | HRV | 480.673 |
| ARE | OMN | 30.021 |
| ARE | THA | 3.600 |
| ARG | BRA | 4706.627 |
| ARG | CHL | 0.251 |
| ARG | ESP | 4412.865 |
| ARG | PER | 144.800 |
| ARG | URY | 16816.953 |
| AUS | NZL | 20946.292 |
| AUS | THA | 324.000 |
| BEL | DEU | 764.922 |
| BEL | DNK | 9.471 |
| BEL | GBR | 0.673 |
| BEL | NLD | 115083.721 |
| BEL | SWE | 4.074 |
| BGR | GRC | 103.772 |
| BGR | ROU | 293.099 |
| BGR | TUR | 10.000 |
| BHR | SAU | 1681.289 |
| BHS | USA | 248.583 |
| BMU | USA | 4.000 |
| BRA | CHL | 3252.170 |
| BRA | PER | 0.800 |
| BRA | URY | 4.800 |
| BRA | VEN | 1797.971 |
| BRB | GRD | 10.515 |
| BRB | TTO | 10.200 |
| BRB | VCT | 3.900 |
| CAN | DNK | 464.600 |
| CAN | GBR | 26.400 |
| CAN | ISL | 31.584 |
| CAN | THA | 0.200 |
| CAN | USA | 1154408.262 |
| CHL | CHN | 5042.000 |
| CHL | COL | 43.000 |
| CHL | ECU | 1210.773 |
| CHL | KOR | 10.800 |
| CHL | PER | 16550.200 |
| CHL | URY | 0.400 |
| CHN | ECU | 3562.400 |
| CHN | IDN | 522.200 |
| CHN | JPN | 235735.600 |
| CHN | KOR | 132433.201 |
| CHN | MEX | 2337.400 |
| CHN | MYS | 3.600 |
| CHN | NLD | 156.400 |
| CHN | NZL | 891.200 |
| CHN | PER | 13383.400 |
| CHN | PHL | 209.800 |
| CHN | RUS | 93356.000 |
| CHN | SEN | 692.600 |
| CHN | THA | 9939.600 |
| CHN | USA | 6918.320 |
| CIV | ESP | 8087.200 |
| CIV | GHA | 1062.400 |
| CIV | NLD | 1791.000 |
| CIV | SEN | 1923.804 |
| COG | SEN | 62.868 |
| COK | NZL | 1.800 |
| COL | ECU | 149965.835 |
| COL | PER | 15.800 |
| COL | VEN | 2529.200 |
| CPV | ESP | 74.413 |
| CRI | MEX | 2.600 |
| CRI | USA | 46.080 |
| CUB | ESP | 175.600 |
| CYP | GRC | 100.401 |
| DEU | DNK | 548212.024 |
| DEU | GBR | 672.247 |
| DEU | IRL | 7.055 |
| DEU | ISL | 2.267 |
| DEU | ITA | 142.155 |
| DEU | LTU | 223.241 |
| DEU | NLD | 907343.422 |
| DEU | POL | 190449.969 |
| DEU | ROU | 24.004 |
| DEU | RUS | 11888.200 |
| DEU | SWE | 4127.200 |
| DNK | FIN | 867.800 |
| DNK | FRO | 201062.232 |
| DNK | GBR | 107252.347 |
| DNK | GRL | 1300.775 |
| DNK | IRL | 115.667 |
| DNK | ISL | 8520.387 |
| DNK | ITA | 431.268 |
| DNK | LVA | 0.400 |
| DNK | NLD | 28162.289 |
| DNK | POL | 26817.297 |
| DNK | PRT | 0.067 |
| DNK | RUS | 20390.268 |
| DNK | SWE | 812203.134 |
| DOM | USA | 0.400 |
| DZA | ESP | 118.040 |
| DZA | ITA | 15.000 |
| DZA | TUN | 9.973 |
| ECU | ESP | 177221.000 |
| ECU | MEX | 14.400 |
| ECU | PER | 46464.044 |
| ECU | VEN | 21400.524 |
| ESP | GBR | 1782.600 |
| ESP | GRC | 1293.939 |
| ESP | HRV | 38.600 |
| ESP | ITA | 127395.636 |
| ESP | KEN | 20.200 |
| ESP | MAR | 8370.000 |
| ESP | MDG | 3377.680 |
| ESP | MEX | 92.000 |
| ESP | MUS | 16782.315 |
| ESP | PER | 83.000 |
| ESP | PRT | 370579.911 |
| ESP | SEN | 804.200 |
| ESP | SLV | 12925.000 |
| ESP | SWE | 2.667 |
| ESP | SYC | 68732.652 |
| ESP | THA | 6262.800 |
| ESP | TUN | 1456.267 |
| ESP | TUR | 650.733 |
| EST | FIN | 3198.947 |
| EST | LTU | 1516.479 |
| EST | LVA | 22972.774 |
| EST | RUS | 8657.400 |
| EST | SWE | 3824.537 |
| FIN | SWE | 115791.081 |
| FJI | KOR | 16.800 |
| FJI | NZL | 8954.707 |
| FJI | THA | 142.400 |
| FRA | NOR | 540484.800 |
| FRA | TWN | 28401.400 |
| FRO | ISL | 14281.609 |
| FSM | THA | 179.400 |
| GBR | GHA | 17.400 |
| GBR | IRL | 278332.618 |
| GBR | ISL | 93582.887 |
| GBR | ITA | 1.400 |
| GBR | NLD | 32474.918 |
| GBR | POL | 6456.800 |
| GBR | RUS | 58461.655 |
| GBR | SWE | 1956.200 |
| GBR | USA | 648.200 |
| GEO | RUS | 16.200 |
| GEO | UKR | 18.400 |
| GHA | JPN | 11.000 |
| GHA | TGO | 14.600 |
| GRC | ITA | 39967.011 |
| GRC | OMN | 0.013 |
| GRC | PRT | 458.800 |
| GRC | ROU | 11.667 |
| GRC | SVN | 1.688 |
| GRC | TUR | 9024.885 |
| GRD | TTO | 7.407 |
| GRD | USA | 11.600 |
| GRL | ISL | 990.507 |
| GTM | HND | 2.000 |
| GTM | MEX | 2411.000 |
| GTM | NIC | 19.559 |
| GTM | PAN | 37.800 |
| GTM | SLV | 3904.240 |
| GTM | USA | 2.600 |
| GUY | SUR | 0.200 |
| HND | USA | 84.800 |
| HRV | ITA | 8341.636 |
| HRV | SRB | 1.693 |
| HRV | SVN | 1108.487 |
| HRV | SWE | 63.419 |
| IDN | IND | 0.400 |
| IDN | JPN | 23.200 |
| IDN | MYS | 13227.400 |
| IDN | PHL | 3904.000 |
| IDN | THA | 4838.200 |
| IDN | VNM | 54.600 |
| IDN | YEM | 28.800 |
| IND | LKA | 117.800 |
| IND | THA | 6.200 |
| IND | VNM | 339.800 |
| IRL | NLD | 731.559 |
| ISL | LTU | 3068.011 |
| ISL | LVA | 326.733 |
| ISL | NLD | 1070.875 |
| ISL | POL | 1490.023 |
| ISL | PRT | 1214.528 |
| ISL | RUS | 10904.133 |
| ISL | SWE | 57.200 |
| ISL | USA | 20.800 |
| ITA | MAR | 279.000 |
| ITA | NLD | 376.065 |
| ITA | PRT | 2578.285 |
| ITA | SWE | 28.600 |
| ITA | TUN | 9263.653 |
| ITA | TUR | 3969.600 |
| JPN | KOR | 169015.334 |
| JPN | MLT | 5.400 |
| JPN | PHL | 19857.200 |
| JPN | RUS | 287.000 |
| JPN | THA | 33652.600 |
| JPN | USA | 45010.000 |
| JPN | VNM | 4773.000 |
| KEN | TZA | 0.600 |
| KNA | USA | 2.800 |
| KOR | MUS | 0.133 |
| KOR | MYS | 19.800 |
| KOR | PHL | 33208.201 |
| KOR | SEN | 164.200 |
| KOR | THA | 8530.800 |
| KOR | USA | 3434.600 |
| KOR | VNM | 9316.600 |
| LKA | MDV | 115.200 |
| LTU | LVA | 46256.326 |
| LTU | POL | 7.800 |
| LTU | RUS | 1124.000 |
| LTU | SWE | 25699.689 |
| LTU | THA | 2.400 |
| LVA | POL | 910.400 |
| LVA | RUS | 1250.000 |
| LVA | SWE | 8263.934 |
| MAR | MRT | 71.600 |
| MAR | SEN | 1985.766 |
| MDG | MUS | 1426.507 |
| MDV | THA | 24.800 |
| MEX | NIC | 0.200 |
| MEX | PHL | 186.600 |
| MEX | SLV | 575.000 |
| MEX | THA | 1417.800 |
| MEX | USA | 31665.067 |
| MUS | MYS | 44.000 |
| MUS | SGP | 1.600 |
| MUS | SYC | 456.667 |
| MYS | PAK | 0.200 |
| MYS | THA | 1600.400 |
| NAM | ZAF | 1330.479 |
| NIC | PAN | 16.000 |
| NIC | SLV | 15.200 |
| NLD | PRT | 64.459 |
| NLD | RUS | 9509.000 |
| NLD | SWE | 1427.732 |
| NLD | TUR | 394.200 |
| NZL | PHL | 1218.800 |
| NZL | PYF | 15.000 |
| NZL | THA | 29.400 |
| NZL | TON | 8.000 |
| NZL | USA | 0.800 |
| NZL | WSM | 61.067 |
| OMN | SAU | 1.467 |
| OMN | THA | 251.600 |
| OMN | YEM | 578.639 |
| PAK | THA | 7.200 |
| PAN | PER | 105.965 |
| PAN | SLV | 1.200 |
| PER | URY | 4.800 |
| PER | VEN | 800.400 |
| PHL | PNG | 4231.200 |
| PHL | THA | 2063.200 |
| PHL | USA | 3955.760 |
| PHL | VNM | 31.200 |
| PNG | SLB | 6.320 |
| PNG | THA | 118.800 |
| POL | ROU | 344.338 |
| POL | RUS | 89307.000 |
| POL | SWE | 28581.424 |
| POL | UKR | 70.800 |
| PRT | STP | 0.200 |
| PRT | SWE | 50.000 |
| SLB | THA | 2.600 |
| SLV | VEN | 126.400 |
| SUR | TTO | 48.577 |
| TCA | USA | 118.400 |
| THA | USA | 5286.200 |
| THA | VNM | 1396.000 |
| THA | VUT | 385.400 |
| THA | YEM | 76.200 |
| TTO | USA | 4.200 |
| TUN | TUR | 32.680 |
| USA | VNM | 0.400 |
