# Supplementary material for: Quantifying global redundant fisheries trade to streamline seafood supply chains
Source: PLoS One. 2024 Jul 10;19(7):e0305779. doi: 10.1371/journal.pone.0305779 (PMC11236095; doi:10.1371/journal.pone.0305779)
Supplement: S7 Table — (DOCX) [file pone.0305779.s007.docx]

# **Supplementary Material – Kuempel et al.** Quantifying global redundant fisheries trade to streamline seafood supply chains

**Table S7.** List of all species two-way traded, total volume of redundant two-way trade (tonnes), and their IUCN ranking (LC = Least Concern, VU = Vulnerable, NT = Near Threatened, EN = Endangered, DD = Data Deficient, NA = Not Assessed)

| **Taxon name** | **Common name** | **Total redundant trade** | **IUCN** |
| --- | --- | --- | --- |
| Acanthocybium solandri | Wahoo | 6.4000001 | LC |
| Anarhichas lupus | Wolf-fish | 551.389338 | NA |
| Anoplopoma fimbria | Sablefish | 45091.1486 | NA |
| Apostichopus japonicus | Japanese sea cucumber | 151.599999 | EN |
| Brama brama | Atlantic pomfret | 1788.058 | LC |
| Brosme brosme | Tusk | 33.8506666 | NA |
| Cancer borealis | Jonah crab | 260.600006 | NA |
| Cancer magister | Dungeness crab | 42057.0667 | NA |
| Cancer pagurus | Edible crab | 18220.2528 | NA |
| Clupea harengus | Atlantic herring | 2380877.34 | LC |
| Clupea pallasii pallasii | Pacific herring | 64750.2004 | DD |
| Cololabis saira | Pacific saury | 396.200003 | NA |
| Coregonus lavaretus | Common whitefish | 595.244735 | VU |
| Coryphaena hippurus | Common dolphinfish | 3.95866669 | LC |
| Crangon crangon | Common shrimp | 33814.3174 | NA |
| Dicentrarchus labrax | European seabass | 2168.31932 | LC |
| Dissostichus eleginoides | Patagonian toothfish | 1000.13868 | NA |
| Dissostichus mawsoni | Antarctic toothfish | 75.8000001 | NA |
| Engraulis anchoita | Argentine anchoita | 2 | LC |
| Engraulis encrasicolus | European anchovy | 134227.808 | LC |
| Engraulis japonicus | Japanese anchovy | 297.799998 | LC |
| Engraulis mordax | Californian anchovy | 140.4 | DD |
| Engraulis ringens | Anchoveta | 1414.80002 | LC |
| Euthynnus affinis | Kawakawa | 247.400003 | LC |
| Euthynnus alletteratus | Little tunny | 357.782004 | LC |
| Fenneropenaeus merguiensis | Banana prawn | 1425.59999 | NA |
| Gadus macrocephalus | Pacific cod | 68901.7994 | NA |
| Gadus morhua | Atlantic cod | 724473.579 | VU |
| Hippoglossus hippoglossus | Atlantic halibut | 757.842801 | NT |
| Hippoglossus stenolepis | Pacific halibut | 59155.8602 | LC |
| Homarus americanus | American lobster | 65424.1589 | LC |
| Homarus gammarus | European lobster | 3330.0727 | LC |
| Illex argentinus | Argentine shortfin squid | 5728.64939 | LC |
| Illex illecebrosus | Northern shortfin squid | 124.672667 | LC |
| Katsuwonus pelamis | Skipjack tuna | 619533.395 | LC |
| Larimichthys polyactis | Yellow croaker | 27080.0001 | LC |
| Lepidorhombus whiffiagonis | Megrim | 8261.57133 | LC |
| Lethrinus lentjan | Pink ear emperor | 2.40000013 | LC |
| Limanda ferruginea | Yellowtail flounder | 2.13333338 | VU |
| Limanda limanda | Dab | 1921.67941 | LC |
| Lophius piscatorius | Angler | 8103.90604 | LC |
| Macrourus berglax | Onion-eye grenadier | 129.67 | NA |
| Makaira indica | Black marlin | 2.96466663 | DD |
| Mallotus villosus | Capelin | 639.800001 | NA |
| Melanogrammus aeglefinus | Haddock | 37038.9521 | VU |
| Melicertus kerathurus | Caramote prawn | 917.088056 | NA |
| Merlangius merlangus | Whiting | 3775.43867 | LC |
| Merluccius hubbsi | Argentine hake | 16279.2527 | NA |
| Merluccius merluccius | European hake | 35339.1084 | LC |
| Merluccius productus | North Pacific hake | 247650.449 | LC |
| Merluccius senegalensis | Senegalese hake | 299.426669 | EN |
| Micromesistius poutassou | Blue whiting | 0.10674 | NA |
| Microstomus kitt | Lemon sole | 184.100728 | LC |
| Molva dypterygia | Blue ling | 6.05326662 | LC |
| Molva molva | Ling | 7748.86203 | NA |
| Mytilus edulis | Blue mussel | 524.985471 | NA |
| Mytilus galloprovincialis | Mediterranean mussel | 6.19993365 | NA |
| Nephrops norvegicus | Norway lobster | 66568.5545 | LC |
| Octopus vulgaris | Common octopus | 40680.8905 | LC |
| Oncorhynchus gorbuscha | Pink salmon | 188306.891 | NA |
| Oncorhynchus keta | Chum salmon | 4334.26671 | NA |
| Oncorhynchus nerka | Sockeye salmon | 106430.91 | LC |
| Pagrus auratus | Squirefish | 950.000001 | LC |
| Pagrus pagrus | Common seabream | 1258.25733 | LC |
| Palaemon serratus | Common prawn | 21.3106669 | NA |
| Pandalus borealis | Northern prawn | 21181.0354 | NA |
| Pandalus montagui | Aesop shrimp | 0.38666669 | NA |
| Panulirus argus | Caribbean spiny lobster | 495.982668 | DD |
| Panulirus gracilis | Blue spiny lobster | 12.4346668 | DD |
| Panulirus longipes | Longlegged spiny lobster | 10.7333336 | LC |
| Parapenaeus longirostris | Deepwater rose shrimp | 6070.7653 | NA |
| Pecten maximus | Great Atlantic scallop | 5.93333343 | NA |
| Penaeus monodon | Giant tiger prawn | 8.80000001 | NA |
| Penaeus semisulcatus | Green tiger prawn | 1678.88933 | NA |
| Placopecten magellanicus | American sea scallop | 40065.2595 | NA |
| Pleuronectes platessa | European plaice | 45918.0389 | LC |
| Pollachius pollachius | Pollack | 73071.1219 | LC |
| Pollachius virens | Saithe | 103772.739 | NA |
| Reinhardtius hippoglossoides | Greenland halibut | 10948.3339 | NT |
| Salmo salar | Atlantic salmon | 4374.43066 | LC |
| Salmo trutta trutta | Sea trout | 511.537326 | LC |
| Sarda sarda | Atlantic bonito | 2383.43655 | LC |
| Sardina pilchardus | European pilchard | 154297.503 | LC |
| Sardinella longiceps | Indian oil sardine | 380.533333 | LC |
| Sardinops sagax | South American pilchard | 19158.8318 | LC |
| Scomber japonicus | Chub mackerel | 590050.57 | LC |
| Scomber scombrus | Atlantic mackerel | 591269.959 | LC |
| Scyliorhinus canicula | Smallspotted catshark | 16.0052804 | LC |
| Sebastes alutus | Pacific ocean perch | 57455.9767 | NA |
| Sebastes marinus | Ocean perch | 636.794799 | NA |
| Sebastes mentella | Deepwater redfish | 1116.54669 | LC |
| Sepia officinalis | Common cuttlefish | 3729.79605 | LC |
| Solea solea | Common sole | 86589.4238 | DD |
| Spisula solidissima | Atlantic surf clam | 42208.0425 | NA |
| Sprattus sprattus | European sprat | 180828.433 | LC |
| Squalus acanthias | Piked dogfish | 8291.30663 | VU |
| Thunnus alalunga | Albacore | 114058.036 | LC |
| Thunnus albacares | Yellowfin tuna | 119286.967 | LC |
| Thunnus obesus | Bigeye tuna | 52790.3133 | VU |
| Thunnus thynnus | Atlantic bluefin tuna | 52.3433349 | LC |
| Thunnus tonggol | Longtail tuna | 1189.68801 | DD |
| Todarodes pacificus | Japanese flying squid | 28.3999994 | LC |
| Todarodes sagittatus | European flying squid | 77.0726675 | LC |
| Trachurus capensis | Cape horse mackerel | 31.3173334 | LC |
| Trachurus japonicus | Japanese jack mackerel | 1441.99999 | NT |
| Trachurus mediterraneus | Mediterranean horse mackerel | 4.71993345 | LC |
| Trachurus murphyi | Inca scad | 44242.8066 | DD |
| Trachurus trachurus | Atlantic horse mackerel | 157332.392 | VU |
| Trachysalambria curvirostris | Southern rough shrimp | 9428.79996 | NA |
| Trichiurus lepturus | Largehead hairtail | 34336.7997 | LC |
| Urophycis chuss | Red hake | 11.4060001 | NA |
| Urophycis tenuis | White hake | 18.3460667 | NA |
| Xiphias gladius | Swordfish | 35771.2062 | NT |
| Xiphopenaeus kroyeri | Atlantic seabob | 0.2 | NA |
